# Verifiable biology

**DOI:** 10.1098/rsif.2023.0019

**Published:** 2023-05-10

**Authors:** Savas Konur, Marian Gheorghe, Natalio Krasnogor

**Affiliations:** ^1^ Department of Computer Science, University of Bradford, Richmond Building, Bradford BD7 1DP, UK; ^2^ School of Computing Science, Newcastle University, Science Square, Newcastle upon Tyne NE4 5TG, UK

**Keywords:** modelling, verification, computational biology, synthetic biology, biological systems, biochemical networks

## Abstract

The formalization of biological systems using computational modelling approaches as an alternative to mathematical-based methods has recently received much interest because computational models provide a deeper mechanistic understanding of biological systems. In particular, formal verification, complementary approach to standard computational techniques such as simulation, is used to validate the system correctness and obtain critical information about system behaviour. In this study, we survey the most frequently used computational modelling approaches and formal verification techniques for computational biology. We compare a number of verification tools and software suites used to analyse biological systems and biochemical networks, and to verify a wide range of biological properties. For users who have no expertise in formal verification, we present a novel methodology that allows them to easily apply formal verification techniques to analyse their biological or biochemical system of interest.

## 1. Introduction

The formalization of biological systems using *computational modelling* approaches as an alternative to mathematical-based methods has recently received much interest. This is so for at least two reasons: (i) computational approaches provide a deeper mechanistic understanding of biological systems and (ii) they are more amenable to easier communication between the biologist and modeller. In particular, ‘formalisms where molecular populations and interactions are modelled as discrete entities and events’, collectively known as *executable biology* [1], or *algorithmic systems biology* [2], enable a more agile co-creation of models across different research domains.

Various computational formalisms have seen a resurgence of use in biology, including process algebra [3,4] symbolic modelling [5], cellular automata [6], knowledge-based expert systems [7], Petri nets [8], membrane computing [9,10] and agent-based systems [11,12]. While these approaches have been useful for modelling and simulating system dynamics [3–7,12,13], conventional simulation-based validations provide only a limited insight for understanding the functional properties and they do not offer assurances about system behaviour [14].

Formal methods have been used in analyses of computational (or executable) biological systems to validate the system correctness and obtain important information about system behaviour, which can be considered as a complementary approach to standard computational techniques. *Formal verification* is a method that *exhaustively* analyses *all* possible system behaviours to evaluate the correctness of a system. Formal verification provides more insight into a natural system than standard methods, e.g. simulation and testing, allowing us to infer ‘more novel information about system properties’ [15].

*Model checking* [16], an *algorithmic* approach to verification, is a computational technique that attempts to verify whether a model with a finite structure satisfies certain properties. Model checking requires a formal model of the system and formal specification of the property, expressed in a logical notation [17–22]. It then evaluates the formal specification against all possible behaviours of the system, which are computed by enumerating all the possible sequences of traces.

Model checking has been widely used in computing and engineering applications in the last two decades to verify various systems, e.g. safety-critical systems [23], concurrent systems [24], distributed systems [25], network protocols [26], stochastic systems [27], multi-agent systems [28,29], pervasive systems [30–32], swarm robotics [33,34] and some engineering applications [35–37]. Due to its novel features to infer information about system behaviour, there is growing interest in applying this technique in systems biology [38,39]. Model checking has been applied to the analysis of various biological systems such as ERK/MAPK or FGF signalling pathway [40–42], EGFR pathway [43,44], T-cell receptor signalling pathway [45–48], cell cycle in eukaryotes [49,50], cell cycle control [51–55], mammalian cell cycle regulation [56,57], apoptosis network [58,59], bladder tumorigenesis [60], quorum sensing [61–63], biological control mechanisms [64], DNA computing [65–67], genetic oscillator [68,69], genetic Boolean gates [61,70–73] and switches [73–75].

Some studies have evaluated the methods used in modelling biological and biochemical networks [1,2,76–81] and the use of formal methods in systems biology [69,82–86]. However, these studies cover only limited and very specific methods, which are usually context-dependent. In addition, modelling and verification aspects are discussed separately from a theoretical perspective with very little attention to the usability and practicality aspects, resulting in very limited benefits to biologists, actual stakeholders and ultimate beneficiaries of the application of formal techniques to the life sciences.

In this paper, we provide a comprehensive overview of the computational models, formal verification and software tools used in model checking of biological and biochemical systems with a holistic view. Because model checking is a very technical concept that is not very well understood by biologists, we provide an illustrative summary of the concept. We also present some guidance derived from our exhaustive survey for non-expert users on how to apply model checking in the formal analysis of biological systems.

The target audience of this paper is mainly biologists and bioinformaticians who have experience of using computational modelling and *in silico* software tools to understand biological processes, systems and complex biological phenomena. For a description of the terms used in this paper, we refer the reader to the Glossary section in the electronic supplementary material.

### 1.1. Significance and contributions

Our work improves previous studies in several dimensions: we (i) provide a wider and up-to-date survey on the most frequently used computational modelling approaches in computational biology; (ii) compare the most used formal verification techniques for analysing biological systems and biochemical networks; (iii) categorize standalone tools and software suites that integrate third-party model checkers and compare a number of software tools; (iv) report an extended updated list of biological systems verified for a wide range of biological properties; (v) provide a comprehensive summary for model checking, which is not available in most review papers, using explanatory figures to explain the core concepts intuitively; (vi) generate a list of property *patterns* recurring in many papers; and (vii) provide an original methodology and guidance for non-expert users on how to apply model checking in the formal analysis of their systems.

The remainder of this paper is organized as follows: §2 summarizes the concept of model checking. Sections 3 and 4 provide an overview of the computational models and software tools used in model checking biological systems, respectively. Section 5 presents the most common property patterns used in systems biology. Section 6 presents our methodology for using model checking when formally analysing biological systems. Section 7 presents some discussions. Section 8 draws conclusions and presents our future work.

## 2. An introduction to model checking

Model checking [16] is an algorithmic verification method that permits the analysis of whether a finite system model satisfies a logical property. Models are usually represented by a compact structure, such as a state transition system or finite state automaton, and logical properties are usually represented by a *temporal logic* formula. The model checking problem consists of verifying whether the temporal logic formula is satisfied by the model in question. This is performed by exhaustively searching the entire state space represented by the model.

If we explain this within the context of a biological system, a state generally refers to the condition or configuration of the system at a given time, based on its biochemical properties. For example, in the context of a cell, its state could refer to the level of gene expression, or the concentration of certain proteins or metabolites. A typical model checking exercise would then be to query the model that represents the biological system in question using some logical specifications, such as ‘if the expression of certain proteins exceeds a desired threshold’, which is expressed as a temporal logical formula.

To formally analyse a biological system using model checking, we need a formal model of the system and a formal specification of the property to be checked. In other words, both system models and temporal logic formulae must be provided in a dedicated syntax that is accepted by model checkers. So, by mapping a biological system into a formal model and translating an informal requirement into a formal representation, we can infer important knowledge regarding system behaviour.

**Figure 1.**
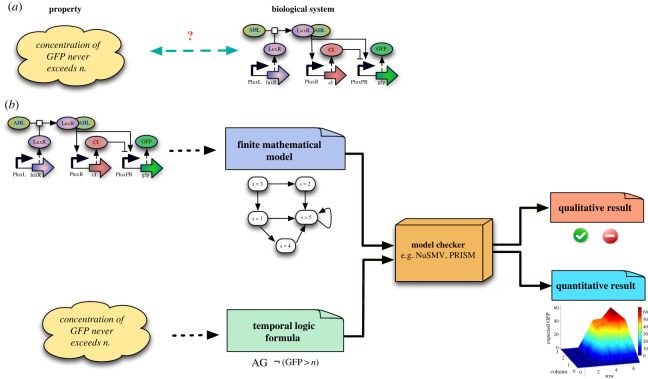
(*a*) We have a biological system and we want to check if the property ‘*GFP never exceeds *n* mature fluorescing proteins*’ is satisfied by the model (Here, GFP represents green fluorescent protein, and *n* presents the level of concentration). (b) Model checking requires two inputs: a formal model of the system, which is a finite mathematical model, and a formal specification of the property, which is a temporal logic formula. Here, the formula is expressed as AG ¬(GFP>n), where ‘G ¬’ represents ‘never’ and ‘A’ represents all execution traces. A model checker (e.g. NuSMV and Prism) then automatically verifies if the specification is satisfied by the model. Depending on the property type, the model checker either produces a qualitative answer (a ‘yes’ or ‘no’), or a quantitative result.

We now give an example to show how model checking works. Assume that we have a biological system and we would like to check a property. To verify whether the system satisfies this property, we need to provide a formal model of the system in the form of a finite mathematical model, and a formal specification of the system in the form of a temporal logic formula. A model checker (e.g. NuSMV [87]) then automatically verifies whether the specification is satisfied by the model. If the model satisfies the specification, then the model checker returns ‘yes’; otherwise, it returns ‘no’ and produces a ‘counter-example’ to help modellers fix any error in their models. Importantly, these counter-examples are sometimes non-obvious or counterintuitive and may guide biologists to formulate new experimental hypotheses to prove their system in the laboratory. Some model checkers support quantitative properties and can produce quantitative results. The overall process is illustrated in figure 1.

Classical model checking is generally used to analyse *qualitative* properties, because model checkers employ temporal logics as a specification language. However, most realistic systems, including biological systems and biochemical reactions, are probabilistic in nature. In addition, to infer more detailed information from the system behaviour, both *qualitative* and *quantitative* analyses are required.

*Probabilistic model checking* is a stochastic extension of classical model checking complemented with quantitative techniques to verify properties about the *likelihood* of the observation of certain behaviour, such as ‘*what is the probability that the expression (i.e. transcription & translation) of a green fluorescent protein (*GFP*) results in n mature and active (i.e. fluorescing) proteins (*GFP* > *n*)?*’ Probabilistic model checkers work similarly to the mechanism shown in figure 1 [27]. However, they require a *probabilistic state machine* (such as *discrete-time Markov chains (DTMCs)* and *continuous-time Markov chains (CTMCs)*) in a dedicated syntax. A probabilistic model checker automatically verifies whether the system model satisfies the property using analytical methods. The most widely used probabilistic model checker is Prism [88].

Although model checking is very useful for system analysis, it has a major drawback: the *state explosion* problem, meaning that the state space grows so quickly that model checking cannot remain scalable. This is especially the case for large models because model checkers exhaustively search this large state space.

*Statistical model checking* [89] is a simulation-based model checking, where ‘the system is simulated finitely many times to generate execution runs and a statistical evidence is obtained for the verification of a property’ [90]. Because the state space is partially explored, the performance is increased but at the cost of precision. Although probabilistic model checking calculates precise results using analytical methods, statistical model checking provides an approximate result based on a confidence interval. Due to its advantage on scalability, statistical model checking has recently gaining popularity.

### 2.1. Property specification

In model checking, properties must be expressed in a suitable *logic*, a formal language describing statements whose truth values change over time. In this section, we discuss some logics used by different types of model checkers.

#### 2.1.1. Temporal logics

Temporal logics are useful specification languages because they can state how the behaviour of systems evolves over time without explicitly referring to time. Namely, a reference to time is done implicitly with the use of *temporal operators*, which quantify over states along an execution path of the system model. These operators are **X** (meaning in the *next* state), **F** (meaning in some *future* state), **G** (*globally* in all future states) and **U** (meaning *until* some future state).

Temporal logics are often classified as *linear* and *branching* temporal logics based on the structure of time, i.e. the underlying time is either linear or branching. *Linear temporal logic* (ltl) [91] and *computation tree logic* (ctl) [92] are the most widely used linear and branching logics, respectively. ltl can express the properties of linear sequences of states. ltl formulae are constructed using the temporal operators **X**, **F**, **G** and **U**. As an example, assume we want to formulate the property ‘GFP never exceeds *n* mature fluorescing proteins’ using the linear time semantics. This property is expressed in ltl as follows:

G ¬(GFP>n).

The underlying time in branching temporal logics is a tree-like structure. Namely, each time point (or state) is followed by several immediate successor time points (states). In addition to temporal operators, branching time logics also employ two *branch quantifiers*, which quantify over execution paths of the system model. These are **E** (meaning *for some* branches, i.e. execution paths) and **A** (meaning *for all* branches). In ctl, temporal operators and branch quantifiers are used together to construct ctl formulae, **EX**, **EF**, **EG**, **E (U)**, **AX**, **AF**, **AG** and **A (U)**. Assume that we want to express the property above using the branching semantics. We can then express this in ctl as follows:

AG ¬(GFP>n).

**Figure 2.**
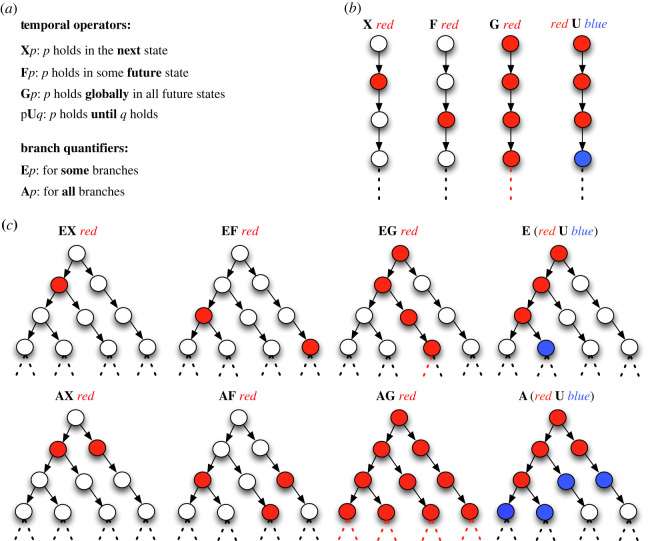
(*a*) Temporal operators (**X**, **F**, **G** and **U**) and branch quantifiers (**E** and **A**). (*b*) Formulae of linear time logics (e.g. ltl) are constructed using temporal operators. ltl formulae intuitively represent linear sequences of states. (*c*) Formulae of branching time logics (e.g. ctl) are built using both temporal operators and branch quantifiers. ctl formulae intuitively represent tree-like ordering of states.

Figure 2 summarizes all the ltl and ctl operators and their informal semantics.

The temporal operators discussed above are the qualitative operators. Some quantitative variants of these operators have also been introduced. *Bounded* ltl (bltl) [91] is a variant of ltl that extends temporal operators with time bounds. For example, the operator **F**^≤*k*^ means ‘in some future state occurring within *k* time units’.

In LTL and CTL model checking, both deterministic and non-deterministic models can be used to represent systems, depending on the nature of the system being modelled. Typically biological models, represented as a state machine, are non-deterministic, as the system being modelled may have multiple possible paths depending on the choices made at each step. To verify a temporal property in a non-deterministic model, the model checker must check all possible paths to ensure that the property holds for all of them. For example, in CTL, to reason about a logical property of the next state in a non-deterministic system, one needs to consider all the possible next states and check whether the property holds for all of them. This can be done using the universal and existential quantifiers to express logical properties that hold for all or at least one of the possible next states (figure 2).

#### 2.1.2. Probabilistic temporal logics

**Figure 3.**
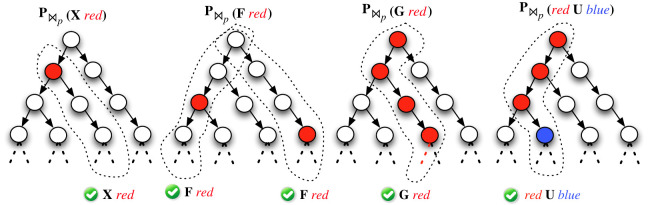
Intuitive meanings of pctl formulae are presented. $P_{⋈p}\;[\varphi ]$ informally means that the probability of taking a branch satisfying φ meets ⋈p, where φ is either **X** *red*, **F** *red*, **G** *red* or *red* **U** *blue*.

A probabilistic temporal logic is a probabilistic extension of a temporal logic. In branching semantics, the branch operators are replaced with a *probability* operator. pctl [93] is a probabilistic extension of ctl, where the **E** and **A** branch operators are replaced with the probability operator $P_{⋈\;p}$. The formula $P_{⋈\;p}\;[\varphi ]$ informally means that the probability of taking a branch satisfying φ meets ⋈p (where ⋈ ∈{<,>,=,≤,≥} is a relational operator and *p* ∈ [0, 1] is a probability). **E** and **A** can be formulated in pctl as **P**_>0_ and **P**_≥1_, respectively (see figure 3). We can express this as follows:

$$P_{\geq 1}\;[G\;\neg (\mathrm{GFP}>n)].$$

Similar to ctl, probabilistic extensions of ltl and bltl are defined, called pltl [94] and pbltl [95], respectively. Both pltl and pbltl extend the temporal operators with a probabilistic operator, interpreted over linear time points.

The probabilistic logics pctl, pltl and pbltl allow the specification of properties for discrete-time systems, e.g. DTMCs, but cannot be used for continuous-time systems. *Continuous stochastic logic* (csl) [96] is a stochastic extension of ctl, which can specify the stochastic behaviour of continuous-time systems, e.g. CTMCs. csl can express *transient* and *steady-state* properties.

## 3. Modelling approaches for biological and biochemical systems

In this section, we provide an overview of some of the most used mathematical and computational modelling approaches (table 1) for analysing biological systems and biochemical networks.

**Table 1.** Modelling approaches that are most commonly used to analyse biological systems and biochemical networks.

modelling approach	formalism
ODEs	XS-systems, piecewise linear, piecewise multi-affine
state transition systems	Kripke structures, Boolean networks, multi-valued logical formalism, reactive modules, statecharts, live sequence charts
rule-based systems	P systems, rewriting systems, pathway logic
Petri nets	stochastic, coloured, discrete, continuous Petri nets
process algebra	stochastic *π*-calculus, Bio-PEPA, BioAmbients

### 3.1. Mathematical modelling approach: ordinary differential equations

The mathematical modelling approach has a long tradition inherited from various disciplines, including genetics, biochemistry, evolutionary biology and systems biology, to understand system dynamics and characteristics [97]. Until recently, coupled *ordinary differential equations (ODEs)* were commonly used as a modelling approach for complex biological and biochemical systems.

In biological modelling, an ODE represents the rate of change of a biological variable with respect to time. A biological variable can represent a wide range of quantities, including the concentration of a chemical species, or the amount of a particular protein in a system. A key assumption in an ODE model is that variables are *continuous*, i.e. concentrations change *continuously* over time, and the system model is deterministic, i.e. it results in precise solutions. This assumption becomes valid when the concentrations are sufficiently high, with an approximate lower bound of 10^3^ molecules, and the reactions are sufficiently fast [98]. This assumption cannot be considered any more, when lower concentrations are considered.

Biological systems have also been modelled using approximation methods, e.g. piecewise linear and *piecewise multi-affine* differential equations. ODEs can be rewritten in some canonical forms: *S-system* is a canonical form in which each differential equation is described in such a way that the concentration change of a product is expressed in terms of the concentration changes of its reactants [99]. An *XS-system* is a form containing a list of expressions describing the concentration change of molecular species and a set of equations that represent some constraints regarding the model parameters [99].

ODEs have been predominantly solved using mathematical tools e.g. Matlab and Cvode. Many biological systems have been modelled using this approach. In order to infer more information about the system dynamics, various tools have been deployed to analyse ODEs using formal techniques. For example, there are model checking tools available allowing formal analysis of certain ODEs: gna [100] (piecewise linear), RoVerGeNe [101] (piecewise linear and piecewise multi-affine) and Simpathica/Xssys [99] (XS-system). These tools have been used in the formal analysis of several biological systems, e.g. repressilator [99], purine metabolism [99], initiation of sporulation in *Bacillus subtilis* [102], quorum sensing in *Pseudomonas aeruginosa* [103], nutritional stress response in *Escherichia coli* [104], onset of the pathogenecity in *Erwinia chrysanthemi* [105], synthetic transcriptional cascade [101] and carbon starvation response of *E. coli* [100,106,107].

There are several other approaches in mathematical modelling applied to biological systems such as stochastic differential equation models in systems biology [108], partial differential equations for biological transport models [109], and algebraic approaches for ODE models that do not require parameter estimation [110]. For a detailed survey on mathematical modelling in biological and biochemical networks, we refer the reader to [77].

### 3.2. Computational modelling approach

A different formalism based on computational models as an alternative to mathematical approaches has been investigated more intensively in recent years. Computational models are an algorithmic or mechanistic approach that uses computation to model and analyse complex systems. While mathematical models rely on equations to describe quantities and their relationships over time, computational models present the system in a rather operational way, as a sequence of steps, or an algorithm that can be executed [1]. The process of creating a computational model entails generating the components of the system, their relationships, and the rules that govern the behaviour of the system. This information is then used to design an algorithm(s) that simulates the behaviour of the system under different conditions.

Two terms have been coined in connection with these models: *executable/computational biology* [1] and *algorithmic systems biology* [2,78], where molecular populations and interactions are modelled as discrete entities instead of continuous events. Computational models come with a set of domain-specific languages in modelling, that are largely used in programming analysis, simulation and verification.

In the following, we review the most popular computational models and their capabilities.

#### 3.2.1. State transitions systems

A (discrete) *state system* is a simple computational model that describes the dynamic behaviour of a system. A state system is composed of a set of finite states and behaviours, and it defines how certain changes in input events cause output events to occur. *State transition systems* comprise a finite set of *states*, representing the states in which the system can be, and a finite set of *transitions*, representing the conditions to traverse between these states. The machine moves from one state to another when an event or condition in the corresponding transition holds. In a state transition system, transitions can also be labelled. Depending on the context, a label can be used for different purposes such as a condition to trigger the transition, an action to perform when the transition is taken, and a probability denoting the likelihood of taking the transition.

**Kripke structures** are simple state transition systems that describe the dynamic behaviour of a system. In a Kripke structure, a state represents a snapshot of the system, a transition represents how the system state evolves, and a path represents a computation of the system.

**Boolean networks** [111] are ‘Boolean state transition systems’ representing gene interactions as ‘a directed graph, where each node represents a gene that is either active or inactive’, edges represent positive or negative regulation, and Boolean functions represent gene expressions. Boolean networks are deterministic, and have a fixed topology. Therefore, the initial configuration does not change with time. Boolean networks are particularly useful when the system data are incomplete. In such cases, we can easily construct a model using only topological information and necessary binary relationships. Therefore, they are often selected as a modelling approach ‘for their amenability to analysis rather than realism’ [4].

Probabilistic Boolean networks (PBN), coupling rule-based representation with probability, provide a suitable framework for large-scale modelling of biological networks with uncertainty and have been used for the study of the topology and dynamic aspects of biological systems [112].

**Thomas’ multi-valued logical formalism** [13,113] is a multi-valued state-transition system, providing a *discrete* modelling framework for regulatory networks. It can also be considered as ‘an abstraction of a special case of piecewise linear differential equations or as a generalization of a restriction of Boolean networks’ [82]. Thomas’ regulatory network models are represented by a labelled directed graph, where a vertex denotes variables representing biological entities (e.g. genes and proteins) and a directed edge denotes abstract interaction between variables. Each edge is labelled with a discrete *threshold* representing the maximum concentration level and a sign ‘+’ representing activation and ‘–’ representing inhibition. A regulatory network is associated with a(n) (a)synchronous state graph denoting the dynamics of the network [113].

**Reactive modules** [114] define a language that provides a compact representation of state transition systems by dividing them into a set of *modules*. A module comprises a collection of local variables and a set of transitions that represent the behaviour of the variables over time. The behaviour of a reactive module is represented by *guarded* transitions, which specify that if the guard is true, then the transition is carried out by performing a certain action and updating the local variables.

**Statecharts** [115] are qualitative but fine-grained state-based transition diagrams used to model the mechanism underlying the system behaviour. Statecharts model the dynamic behaviour using states and events that trigger transitions between states. Statecharts permit modelling at multiple levels by allowing states to be composed of substates, which can be ‘zoomed in’ and ‘zoomed out’. States can also be divided into ‘orthogonal states’; thus, concurrency can be described [116].

**Live sequence charts (LSCs)** [117], an extension of message sequence charts, are a scenario-based approach for analysing system behaviour. Live sequence charts formalize various scenarios of behaviours between different system elements, e.g. ‘required’, ‘possible’ and ‘forbidden’ scenarios.

#### 3.2.2. Rule-based systems

Rule-based systems model the state of the system as a molecular species and state changes as molecular interactions specified by rules.

**Rewriting systems** contain sequences of discrete steps in which a subterm of a formula is replaced by another [118]. Rewriting systems comprise a set of objects and rewrite rules that represent the transformation of these objects. In systems biology context, a rewriting rule defines ‘a step in a biological process such as metabolism or intra/inter-cellular signalling’ and describes ‘the behaviour of proteins and other components depending on modification state and biological context’ [5].

**Pathway logic** [43] is a rewriting systems-based ‘algebraic structure’ enabling the building and analysing network models of biological processes, where system states and behaviour are represented by algebraic structures and rewriting rules, respectively. Pathway logic consists of ‘data types representing cellular components such as proteins, small molecules, complexes, compartments/locations protein state and post-translational modifications’ [5]. Pathway logic permits qualitative analysis, e.g. ‘static and dynamic structure of reaction networks’ but does not permit specifying kinetic constants for reactions; stochastic simulations, therefore, are not supported.

**BioNetGen** [119] is a ruled-based system based on structured building blocks that represent proteins and protein complexes. BioNetGen captures ‘site-specific details of protein–protein interactions’ and provides visualization of these interactions. The language also allows molecules to be combined into complexes through ‘binding sites’. The rules specify the biochemical reactions occurring in the system and can be used to construct bimolecular networks.

**Biocham** [120,121] is another rule-based system comprising three languages, modelling biochemical processes at different levels of abstraction: Boolean (i.e. ‘asynchronous Boolean transition systems’), concentration (i.e. ODEs), and stochastic (i.e. continuous-time Markov chains).

**P systems** are the key models of the *membrane computing* theory [122], which is a ‘branch of natural computing’. These models emphasize the ‘compartmentalized nature of biological systems’. The central element of any P system is a membrane structure consisting of regions (or compartments) containing multiple sets of interacting objects through locally specified rewriting and communication rules [123]. ‘P systems *evolve* by repeatedly applying these rules, mimicking chemical reactions and transportation across membranes’ [124]. The close similarity between the model and key elements of the cellular biology makes it suitable not only for modelling purposes, but it also enhances communication between modellers and biologists or any other wet laboratory experimentalists.

**Other rule-based systems**, including **NFsim** [125], **Kappa** [126] and **little b** [127], have been introduced to address combinatorial explosion in the number of interactions by explicitly providing mechanisms to model coincidental modifications or conformations that need to be represented. In biological models, combinatorial explosion can happen when attempting to count or analyse all possible combinations of biological factors or entities. For example, in genetic engineering, biologists may be interested in all potential gene deletion or mutation combinations to produce a desired phenotype or characteristic.

#### 3.2.3. Petri nets

Petri nets are well-established formalisms describing distributed and concurrent systems behaviours. A Petri net is a graph with two types of nodes: places containing tokens and representing various available resources, and transitions describing events that will fire under certain circumstances. Petri nets can be considered a more general form of Boolean networks because several tokens might fire concurrently at the same time. We also note that while Boolean networks can be executed deterministically, Petri nets can be executed non-deterministically.

There are several variants of Petri nets. **Stochastic Petri nets** [128,129] make use of a stochastic simulation algorithm where transitions with associated rate are fired and a ‘period of time is calculated and added to the global clock’ [98].

**Coloured Petri nets** [130] provide a means to deal with multiple possible values associated with each place and produce a more compact system representation [131]. Petri nets can also be defined in discrete and *continuous* semantics.

#### 3.2.4. Process algebras

Process algebras (process calculi) are ‘a diverse family of related formalisms that describe distributed concurrent processes, such as tasks inside a computer program or a collection of programs’, interacting in accordance with certain communication protocols [98]. *π*-calculus [132] is a model for concurrent mobile processes where the communication topologies evolve dynamically. In the biological models based on *π*-calculus, molecules with binding sites are represented as processes with communication channels. The simulation is similar to that of a standard Petri net and provides a qualitative view of the behaviour of the system.

Several variants of process algebras have been introduced: **Stochastic *π*-calculus** [133] enables ‘quantitative simulations by associating a rate constant with each channel’ and providing means to compute a probability based on this rate [98]. **PEPA** (performance evaluation process algebra) is a different stochastic process algebra used for modelling reagent-centric and pathway-centric approaches [134] and for different signalling pathways [3,134–136] and synthetic biology designs [137]. **Bio-PEPA** [138] is a ‘modification of PEPA, incorporating stoichiometry and the use of kinetic laws in rate functions’, which is adequate for modelling biological systems [98]. **BioAmbients** [139], an extension of the Ambient calculus with stochastic features, as in stochastic *π*-calculus, enables to model biological systems with compartmental nature. **BlenX** [140] is a high-level stochastic process algebra-based textual language that is explicitly designed to model biological entities and their interactions.

Stochastic *π*-calculus has been applied to modelling of biological systems such as a Circadian clock mechanism used in many biological organisms to regulate time-based behaviour [141] and constructing dynamic models for the simulation of gene regulatory networks from simple computational elements [142].

#### 3.2.5. Other computational systems

In addition to the computational models discussed above, other executable modelling formalisms exist for biological and biochemical systems. In the following, we briefly discuss them:

**Systems biology markup language (SBML)** [143] is an XML-based language developed to store biological models and facilitate communication between various software platforms. SBML files contain the program artefacts about species, reactions and compartments, as well as other variables, such as events, units and concentrations.

**Hybrid systems** [144–146] combine both continuous and discrete aspects into a single model. In general, continuous behaviour is represented by (piecewise linear) differential equations, and discrete behaviour is represented by computational models. Hybrid systems provide a means to close ‘the gap between mathematical models and computational models by combining them’ [1]. Some examples of hybrid models include hybrid discrete-continuous systems, hybrid automata [147] and hybrid Petri nets [148].

**Cellular automaton** is a formalism frequently used for modelling biological systems [149], including ‘pattern formation (morphogenesis)’ [6], ‘ecology and population biology, immunology, oscillations, diffusion processes, fibroblast aggregation, ant trails and others’—[150] is an overview of different cellular automata models.

Other two notable approaches are **agent-based systems** [12] and **knowledge-based systems** [7].

### 3.3. Example: gene expression

**Figure 4.**
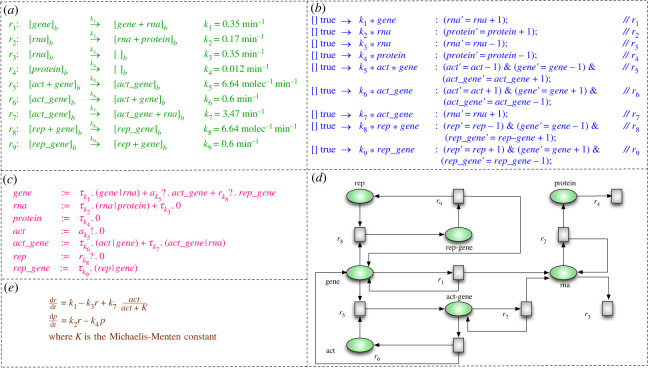
(Redrawn from [151].) A system comprising ‘positive, negative and constitutive expression of a gene’. (*a*) **Rule-based model (P systems).** The system consists of a bacterium represented using a membrane, called *b*. The transcription and translation are represented as multi-set rewriting rules with the associated kinetic constants. (*b*) **State-machine model (reactive modules of Prism).** The model consists of modules, and each module is characterized by a set of commands. A command has the form: $[act]\;g\to \gamma_{1}\;:\;u_{1}+\cdots +\gamma_{n}$, where ‘*g* is a predicate over all the variables of the model’ and each *u*_*i*_ describes a tradition of the module, where the ‘new values of the variables’ are calculated. The expressions *γ*_*i*_ are used to express rates associated with the transitions. The label *act* is used to synchronize commands occurring in different modules. (*c*) **Process algebra model.** The process called *gene* defines ‘all possible interactions consisting of a constitutive reaction, a positive regulation, or negative regulation’. The other processes describe subsequent interactions. (*d*) **Petri net model.** This describes all of the interactions presented above. If a token appears in the place *gene*, then we only describe constitutive expression; if a token is in *gene* and *n* in act with *n* ≥ 1, then we describe a positive regulation; and if we start with ‘one token in *gene* and *m* in *rep* with *m* ≥ 1, then we have negative regulation’. (*e*) **ODE model.** The same interactions are described as a set of differential equations.

Here, we illustrate some of the modelling approaches for a running example, taken from [151]. The biomolecular system comprises ‘*positive*, *negative* and *constitutive* expression of a gene’. The model contains a gene’s specification, its translated protein, the transcribed RNA, and any activator and repressor molecules that bind to the gene to either speed up transcription or block it from being transcribed. When a gene is expressed positively, it means that it is activated in reaction to a particular signal or stimulus, which causes the production of the corresponding protein product. When a gene expresses negatively, it means that it is activated or repressed in reaction to a particular signal or stimulus, which results in minimal or no protein synthesis. A gene that expresses at a constant amount in all cells is said to be constitutively expressed, independent of external signals or stimuli. Figure 4 shows the models of this system using different formalisms.

## 4. Software tools used in verifying biological and biochemical systems

Below we present a comparative summary of important features of the software tools and methods used/developed in this context.

### 4.1. Standalone model checkers

Here, we present state-of-the-art model checkers that are used in systems biology and biochemical networks. An overview of the standalone model checking tools is given in table 2, which provides a summary of the tools and important features.

**Table 2.** Model checking tools used to analyse biological systems.

**Spin** [152] is a popular model checking tool for verifying qualitative temporal properties of various systems. System models to be verified are specified in the Promela language, corresponding to a Kripke structure, and temporal properties are expressed as ltl formulae (or ‘assertions’). Spin also provides an interactive and guided simulator that displays model execution traces. The tool employs a number of methods to improve model checking process and reduce storage space.
**NuSMV** [87] is one of the most widely used model checking tools for verifying the correctness of finite state systems. NuSMV is a symbolic model checker, because it uses a symbolic compact representation of states to reduce the state space. The NuSMV high-level language allows for the writing of modular hierarchical descriptions and reusable components. A NuSMV model corresponds to a Kripke structure. Temporal properties can be expressed in both ltl and ctl.
**Antelope** [153] is a model checking tool for modelling and analysing (branching-time) Boolean networks. The branching time can represent some important phenomena, e.g. ‘asynchrony’, incomplete system information and ‘interaction with the environment’ [153]. To express more Boolean network-specific properties, Antelope employs a ‘*hybrid* extension of ctl, enabling the specification of the set of stable and unstable steady states’ [153]. Rather than simply checking if the given property is true in the initial state, Antelope returns all states that satisfy the given property. This adoption is more preferable in the context of Boolean networks [153]. Antelope is a symbolic model checker, building state space based on compact representations.
**Prism** [88] is one of the most commonly used probabilistic model checking tools for formal modelling and analysis of probabilistic systems. Models are written in a ‘state-based’ high-level language based on ‘reactive modules’. Prism allows the building and analysis of various probabilistic models such as discrete-time Markov chains (DTMCs), continous-time Markov chains (CTMCs) and Markov decision processes (MDPs). It uses ‘compact and structured data structures based on (multi-terminal) binary decision diagrams (BDDs)’ to scale down the size of models. As property language, various probabilistic logics are supported, including ltl, pctl and csl. Prism also provides a ‘discrete-event simulation engine’, supporting statistical model checking, and employs various analysis techniques.
**Plasma Lab** [68] is a statistical model checking tool, deployed for probabilistic systems, e.g. DTMCs and CTMCs. Plasma Lab supports several modelling languages, including reactive modules and a rule-based biological language and some property specification languages, including bltl. The bltl language allows the specification of temporal properties with bounds expressed in steps or time units. Plasma Lab extends bltl with a probability operator, which permits specifying probabilistic properties.
**Mocha** [154] was the first model checker developed to formally analyse reactive modules in a modular and hierarchical manner. The description languages allow one to model systems with synchronous and asynchronous components. In Mocha, properties are expressed as an extension of ctl, called atl. Unlike ctl and ltl, the logic atl provides a selective quantification, which allows one to specify a strategy to reach a desired state. Mocha is a symbolic model checker that employs a BDD engine. The tool also provides a tool support for simulating the reactive systems and displaying the simulation traces in a message sequence chart.
**Maude** [155] is a ‘high-performance reflective language’ based on rewriting systems and an integrated software suite using a range of tools for the modelling and analysis of rewriting systems. Maude models can be translated into various formalisms to conduct various analyses, such as model checking, theorem proving, statistical model checking, debugging, and searching. The Maude model checker is a part of the Maude system that checks whether a rewriting system satisfies a property specified in ltl. The Maude model checker does not use any compact BDD structures to store the states. Maude also provides support for Pathway logic.
**BioLab** [45] is a statistical model checking tool that enables formal verification of BioNetGen models. BioLab simulates models using a BioNetGen simulator, and then verifies properties, written in ‘probabilistic bounded linear temporal logic (pbltl)’, against generated stochastic traces. pbltl is an extension of bounded linear temporal logic (bltl) with a probability operator to bind the likelihood of a bltl formula to hold. BioLab generates as many simulations as needed to verify a property. If more samples are needed to infer whether the property is verified, then BioLab continues to generate more simulation traces until a decision regarding the property has been made.
**mc2** [156] is a statistical model checker based on ‘Monte Carlo approximation’. The tool accepts a set of simulation traces as input rather than a system model specified in a high-level language. Simulation traces can be obtained from any simulation output, e.g. ODEs, CTMCs and Gillespie. As property language, mc2 employs a probabilistic temporal logic with numerical constraints: i.e. pltl with numerical constraints. mc2 can cope with state spaces beyond the current limits of the model checkers that perform exhaustive analyses. Also, the model checking process is generally much quicker because the entire search space is not explored.
**BioDivine** [157] is a set of tools (see [158]) developed for automated analysis of biological interaction networks with respect to properties expressed in various temporal logics. It consists of tools supporting ODE models: BioDivine core (a tool for LTL model checking of piecewise multi-affine ODE models), Pmc (a tool for parameter synthesis of piecewise multi-affine ODE models with unknown kinetic parameters), Pythia (a tool for parameter synthesis of piecewise multi-affine ODE models with respect to hybrid CTL) and Parasim (a tool to analyse the robustness of ODE models with respect to STL and STL* formulae). BioDivine also provides tools for analysis of discrete models—Aeon (a tool for bifurcation analysis of asynchronous Boolean networks) and Parsybone (a tool for the synthesis of logical parameters in regulatory networks—specified in Thomas’ formalism).
**Simpathica** [99] is a set of tools deployed for modelling and analysing biological networks. A network system, e.g. regulatory and signalling, is constructed using a graphical model editor, which allows the description of ODEs (based on XS systems). The system can be analysed using different tools and techniques. The Octave tool enables the simulation of the model to analyse its behaviour. Simpathica also allows analysis of temporal behaviour of biological networks using its back-end model checker xssys. The xssys tool can verify ctl queries, repressing questions about the behaviour of a biological network. These queries are verified against a set of simulation traces. xssys can therefore be considered as an approximate model checker. XSSYS also provides a natural language interface that can be used to construct temporal logic formulae using plain English.
**BioModelAnalyzer** [159] is a web-based toolset developed for the modelling and analysis of gene and protein interaction networks. Based on the Qualitative Networks formalism, users can graphically draw biological models manually or using a built-in library. Users have access to three analysis engines to test their models: standard simulation tools, an interface for the stability testing algorithm and a graphical linear temporal logic (LTL) editor and analysis tool. The tool also allows users to construct LTL properties using a graphical language supported by drag-and-drop features to visually construct queries and evaluate the results. The tool hosts a knowledge base that ‘consists of definitions as well as example usages of LTL operators and developmental end states’.

A comparison of these tools is presented in table 3, where we compare them according to *modelling formalism* (i.e. which modelling approach the tool supports), *specification language* (i.e. logical languages used to express properties), *type* (i.e. model checking method) and *usage* (i.e. for which purpose(s) the tool is used, e.g. *property checking* to verify required properties, *parameter estimation* to find missing parameters in models, and *parameter optimization* to find the optimal set of values).

**Table 3.** Comparison of model checking tools.

software tool	modelling formalism	specification language	type	usage
Spin	Kripke structures	LTL	temporal	property checking
NuSMV	Kripke structures	LTL, CTL	temporal	property checking
Antelope	Boolean networks	CTL (hybrid)	temporal	property checking
Prism	reactive modules	LTL, PCTL, CSL	temporal, probabilistic	property checking
Plasma Lab	reactive modules	BLTL + probability	temporal, probabilistic	property checking
Mocha	reactive modules	ATL	temporal	property checking
Maude	rewriting systems, pathway logic	LTL	temporal	property checking
Biolab	BioNetGen + simulation traces	PBLTL	probabilistic	property checking, parameter estimation
mc2	simulation traces	PLTL + constraints	probabilistic	property checking, parameter optimization
BioDivine	ODEs	LTL	temporal	property checking, parameter synthesis
Simpathica & XSSYS	ODEs	CTL	temporal	property checking
BioModel Analyzer	qualitative networks (GUI)	LTL	temporal	property checking

### 4.2. Software suites employing model checkers

A software suite/platform is different from a standalone tool in that a software suite contains a collection of software applications to perform more than one functionality.

Model checkers have also been integrated and used in software platforms developed to analyse biological systems. Table 4 presents an overview of the integrated software suites that employ model checking tools. In table 5, we compare the software tools similar to those in table 3. In table 5, we also mention the model checking tools employed.

**Table 4.** Software suites integrating model checking tools.

**Play-engine** [160] is a tool developed to build, execute, analyse and verify scenario-based requirements. Behavioural requirements are specified in a visual formalism called *live sequence charts (LSCs)*. The required behaviour is captured by playing and constructing scenarios in a GUI. This process is called *play-in*. The output of the play-in process is then automatically translated into a formal specification visualized as an LSC [161]. In the *play-out* process, these requirements are validated by executing the resulting LSCs. The play-out process is extended with *smart play-out*, which makes use of model checking while executing behavioural requirements. Play-Engine relies on some third-party model checkers, including Smv [162].
**SMBioNet** [163] is a tool for modelling and analysing biological regulatory networks. The description language of the tool is the BioNetGen language based on Thomas’ multi-valued logical formalism, and the property specification language is ctl. SMBioNet allows the selection of models of a biological regulatory network based on certain properties. Namely, temporal properties of the regulatory network are expressed as ctl properties. The tool then generates all corresponding asynchronous state graphs that satisfy these properties. SMBioNet employs NuSMV to perform model checking. Therefore, it uses symbolic model checking approach.
The **Infobiotics workbench (Ibw)** [73,98] is an integrated software toolkit to perform various analyses for a stochastic extension of P systems. The software platform enables modelling, simulation, model checking and parameter optimization using different tools and methods: (i) simulations are performed using either a ‘stochastic simulation or deterministic numerical method using Mcss (a simulator for multi-compartment stochastic P system models)’; (ii) parameter optimization is performed by evolutionary algorithms using POptimizer; (iii) system properties of ‘temporo-spatial behaviour’ are verified using Pmodelchecker; and (iv) all experiments can be visualized using the Infobiotics Dashboard [98].
Pmodelchecker employs the Prism and mc2 tools to perform probabilistic and statistical model checking, respectively. Pmodelchecker supports all the specification languages that these tools support, i.e. ltl, pctl, csl and pltl with numerical constraints. An important feature of Pmodelchecker is that it provides a natural language query tool to assist in constructing properties using natural language statements without a formal syntax.
The **KPWorkbench** [164] is an integrated software toolkit that allows modelling and analysing unified membrane P systems, called kP systems [165,166]. The platform allows the simulation and verification of membrane systems using several native and third-party tools. The framework features a native simulator that permits the simulation of system models written in the kP–Lingua language [124]. In addition, it also integrates an agent-based high-performance simulation environment [70,167]. KPWorkbench’s verification component, supporting both ltl and ctl properties, allows the verification of kP models using the Spin and NuSMV model checkers [168,169]. In order to facilitate the formal specification, KPWorkbench integrates a property language using natural language statements, from which the Spin and NuSMV formulae are translated automatically.
The **Biocham** system [170] is a modelling and analysis platform for rule-based systems. The tool allows modelling biochemical systems, simulating Boolean, differential and stochastic models, and verifying biological properties as well as ‘developing, correcting, completing, and coupling models’. Biocham can estimate missing model parameters from temporal logic properties. The tool can also check that temporal logic properties are not violated during the model building process. In addition, Biocham can ‘automatically search for parameter values that reproduce the specified behaviour of the system under different conditions’ [121]. Biocham employs NuSMV to carry out the model checking task. As property specification language, the tool supports two types of formulae: qualitative properties are expressed in ctl and quantitative properties ‘about concentrations and their derivatives’ are expressed in ltl with numerical constraints.
The **model-checking kit (MCKit)** [171] is a software suite for a collection of tools including model checkers, providing (various variants of) Petri net models with formal analysis and verification, as well as deadlock and reachability checking. The kit can be considered as a common interface for various verification tools. This allows the kit to run different state construction techniques, such as explicit state and symbolic methods. The model checking tools include Spin, smv and LoLA. MCKit accepts both ltl and ctl.
**Bio-PEPA eclipse plug-in & workbench** [138] are two software tools developed to support biochemical networks, based on the Bio-PEPA language. The Bio-PEPA Eclipse Plug-in is a modelling environment that incorporates various features such as a simulator based on the stochastic Gillespie algorithm, static and model coverage analyses, and a GUI to visualize results.
The **bio-PEPA workbench** tool enables modelling and analysing biochemical networks using different techniques, such as stochastic simulation and model checking. The tool maps Bio-PEPA models to different targets (e.g. ODEs, CTMCs and SBML) to use different analysis methods. The Bio-PEPA Workbench does not integrate a model checker directly, but it complies Bio-PEPA models to CTMCs in the reactive modules format accepted by Prism. These modes can then be verified against csl properties.
**Genetic network analyser (GNA)** [100] is a tool developed for qualitative modelling and analysis of genetic regulatory networks given in the form of piecewise-linear ODEs. gna provides a GUI for building, editing and visualizing GRN models. These networks can be analysed using simulation and model checking. The network dynamics can be observed through a qualitative simulation, ‘resulting in predictions adapted to available gene expression data’. The qualitative properties of GRNs are specified in a temporal logic using natural language query templates. These properties can be expressed in ctl and ctrl. ctrl, which subsumes both ctl and ltl, is an extension of ctl with regular expressions (and fairness operators). gna employs NuSMV and Cadp [172] for model checking. gna can export and import from SBML. The tool is also compatible with the Systems Biology Graphical Notation (SBGN) format.
The **Taverna services for systems biology (Tav4SB)** [173] is a web-based software platform to design and analyse kinetics model of biological systems, described as a set of ODEs. Tav4SB accepts ODE models in the SBML format. The services provided by the tool are simulation of the kinetic model using the SBML ODE Solver library, probabilistic model checking of csl formulae using Prism, ‘visualization of data series, such as ODEs trajectories or values of parametrized CSL properties, and probabilistic distribution sampling, using Mathematica’, and ‘high-level analysis, such as multi-parameter sensitivity analysis’ [173].
**RoVerGeNe** [101] is a tool for analysing biological regulatory networks, described as (piecewise multi-affine) ODEs. RoVerGeNe can be used to carry out robustness analysis, meaning that ‘a dynamical property is satisfied by every parameter in a given set and for every initial state in a given region’, and parameter synthesis, meaning ‘searching for valid subsets of a given parameter set’. RoVerGeNe employs the NuSMV model checker, and temporal properties are expressed in ltl.
**Animo** [174] is a toolset for analysing biological pathways, represented by ‘networks of Stochastic Hybrid Automata’. The tool combines Uppaal Stochastic Model Checking, a plugin used by biologists, and SimBiology, a plugin of Matlab to simulate reactions. By integrating translators from SBML (and XGMML) used by ‘Cytoscape and SimBiology to stochastic and hybrid automata’, it allows stochastic model checking analysis techniques for ‘stochastic and hybrid systems’ using Uppaal SMC and the specification formalism of weighted metric temporal logic.

**Table 5.** Comparison of model checking software suites.

software tool	modelling formalism	specification language	third-party tools	type	usage
Play-Engine	live sequence charts	play-in, play-out	smv	temporal	capturing and executing requirements
SMBioNet	multi-valued logical form	CTL	NuSMV	temporal	model selection
Ibw	P systems	LTL, PCTL, CSL, PLTL + constraints	Prism, mc2	temporal, probabilistic	property checking, parameter optimization
Biocham	Biocham (ODEs, stochastic, Boolean)	CTL, LTL + constraints	NuSMV	temporal	property checking, parameter estimation
MCKit	Petri nets	LTL, CTL	smv, Spin, LoLA	temporal	property checking
Bio-PEPA	Bio-PEPA	CSL	Prism	probabilistic	property checking
gna	ODEs	CTL, CTRL	NuSMV, cadp	temporal	property checking
Tav4SB	ODEs	CSL	Prism	probabilistic	property checking, param. sensitivity analysis
RoVerGeNe	ODEs	LTL	NuSMV	temporal	parameter estimation, robustness analysis
Animo	SBML	WMTL	Uppaal-SMC	temporal	property checking

In addition to the most popular software suites presented in table 4, other tools have also been developed, such as U-Check BMS15, Mule [175], PyBioNetFit [53] and Bio-ModelChecker [47].

### 4.3. Examples of biological systems analysed using model checking

In this section, we present biological systems analysed using model checking techniques. Table 6 presents a selected range of systems. An extended list with references is provided in electronic supplementary material, §1.

**Table 6.** Some biological systems and biochemical networks analysed using model checking.

modelling formalism	software tool	biological systems modelled
Kripke structures	Spin	mucus production in *P. aeruginosa*, genetic network of *Arabidopsis thaliana*, quorum sensing, genetic gates, pulse generator
	NuSMV	EGFR network, molecular interaction network of a macrophage, quorum sensing, genetic gates, pulse generator, T-helper cell plasticity
Boolean networks	BooleanNet	abscisic acid, mammalian immune response, T-cell large granular lymphocyte leukaemia, cell cycle gene identification
	Antelope	*A. thaliana* root stem cell niche, flower organ specification, root stem niche
	BooleNet	mammalian cell cycle, yeast cell cycle
	NuSMV	*D. melanogaster* embryo development, budding yeast cell cycle, bladder tumorigenesis
multi-val. logic form	SMBioNet	mucus production in *P. aeruginosa*, biosurfactants production in *Pseudomonas fluorescents*, breast cancer, FGF signalling in *Drosophila melanogaster*
	GINsim	yeast cell cycle, *Drosophila* signalling pathways, MAPK pathway, TCR signalization, mammalian cell cycle
reactive modules	Prism	ERK/MAPK & FGF signalling pathways, codon bias, ribosome kinetics, transient oscillator, bone pathologies, genetic gates, genetic toggle switch
	Plasma	genetic oscillator, simple biochemical system
	Mocha	signalling cross-talk during *C. elegans* vulval development, cell fate specification during *C. elegans* vulval development
P systems	Ibw	cell cycle in eukaryotes, gene expression, liposome logic, quorum sensing, repressilator, pulse generator, genetic gates
	KPWorkbench	quorum sensing, genetic logic gates, pulse generator
rewriting systems	Maude	ERK/MAPK & EGFR signalling networks, molecular interaction network of a macrophage, Rho GTP-binding cycle, signal transduction
pathway logic	Pathway Logic Assistant	MAPK & EGFR signalling networks, HGF/HGFR & IL6/IL6R signalling pathways, response of melanoma cancer cells to drugs, cross-talk in breast cancer
BioNetGen	BioNetGen, BioLab	T-cell receptor signalling pathway, activation of Jak-family protein tyrosine kinases, yeast pheromone response pathway, phosphorylation and scaffolding in MAPK pathways, B-cell antigen receptor signalling, p53-induced apoptosis
Biocham	Biocham	gene expression regulation, cell cycle control, ERK/MAPK, synthetic transcriptional cascade, translation initiation in sea urchin, cell cycle on the mitosis phase, phosphorylation cycles
Petri nets	Prism, mc2, MCKit, Biocham	ERK signal transduction pathway, receptor signalling, kinase cascades, cell-cycle regulation, wound healing, neuronal cell fate decision model in *Caenorhabditis elegans*, angiogenetic process, Wnt/*β*-catenin signalling pathway, DNA walker, quorum sensing
process algebra	Bio-PEPA, BioSPI, etc.	epidemiological models avian influenza, yeast pheromone pathway, circadian clock in *Ostreococcus tauri*, genetic network with a negative feedback, cell growth and damage from cancer treatment, hypertorus communication grid, mumps virus
ODEs	NuSMV	nutritional stress response in *E. coli*, mammalian cell cycle regulation
	BioDivine	transcription in *Bacillus subtilis*, ammonium transport in *E. coli*, genetic regulatory networks, FGFR3 signalling pathway
	Simpathica/Xssys	repressilator, purine metabolism, yeast cell
	gna	gene regulatory networks, nutritional stress and carbon starvation response in *E. coli*
	Tav4SB	enzymatic reaction model
	RoVerGeNe	synthetic transcriptional cascade, toggle switch, two-genes network with stimulus

## 5. A design pattern language for biological verification

When a system is analysed through model checking, users formally specify the requirements that the system in question should satisfy. Formal languages are highly expressive and allow users to write a wide range of properties. However, to obtain a more accurate view of the system behaviour, a system model should be queried with the most appropriate properties to reveal the system dynamics. This makes the property specification at least as important as the system specification and modelling.

Our goal is to identify and discover the most common biological properties and capture properties exhibited by models and/or experiments. To do this, we surveyed case studies addressing the formal analysis of biological and biochemical systems and investigated the properties that are most commonly used. We derived this new property pattern language by mining the literature referred to in electronic supplementary material, §1.

One of our important observations is that, although we have gathered a few hundred properties, most of these properties can be categorized into a small number of *property patterns*. The variety simply comes from different expressions of the same property. Based on this observation, we have constructed a list of property *patterns* for most frequent properties used in biology.

The concepts ‘patterns’ and ‘pattern languages’ were introduced in [176]:

‘Each pattern describes a problem which occurs over and over again in our environment, and then describes the core of the solution to that problem, in such a way that you can use this solution a million times over, without ever doing it the same way twice.’

In many engineering applications, specific types of patterns have been identified using natural language patterns [177–179]. To capture biological properties, in [15] we introduced a pattern language whose primitive elements were patterns. These patterns offer a comprehensive set of templates that help users easily generate formal properties. Table 7 presents a subset of this language. In order to facilitate the property building process, the natural language query (NLQ) tool, developed in [15], constructs properties from a predefined set of property patterns. Using a graphical user interface, the user selects a pattern in the form of a predefined natural language statement and provides the necessary values (e.g. concentration amounts and time bounds), then the tool automatically converts the pattern to its formal counterpart based on the target model checker selected.

**Table 7.** Property patterns for biological properties.

pattern	informal meaning	example
existence	a property eventually becomes true	the concentration of *S* becomes greater than the concentration of *F* [180]
absence	a property becomes never true	it is not possible to activate *X* in any pathways [181]
universality	a property is always true	*GTP* level is always less than *k* [99]
until	a property is true until another property becomes true	the protein *A* degrades before binding to the protein *B* [41]
response	a property is followed by another property	if reaction *R* is possible, then eventually the reaction *R* happens [43]
steady-state	a property becomes true in the steady-state	in the long run, *FGF* is free [182]
oscillation	a property is true infinitely often	oscillation terminates in species *X* ∈ {*A*, *B*, *C*} [183]
reward	expected reward at/within certain time	expected time to reach a state in which all gates finished executing [184]

**Figure 5.**
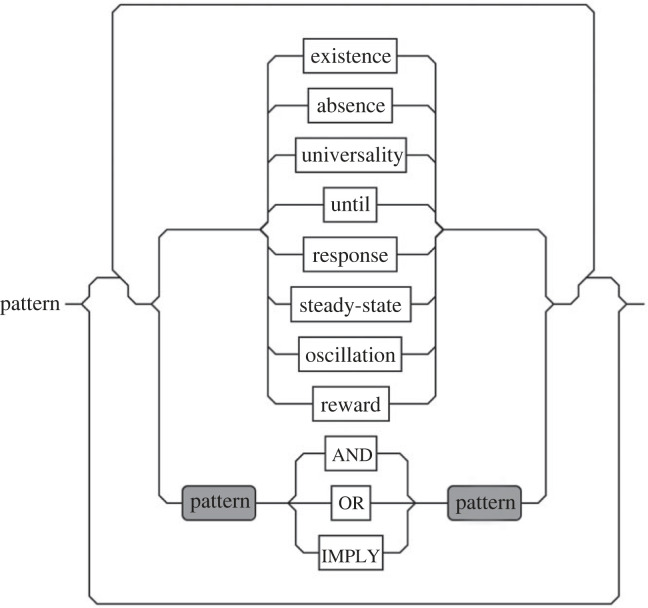
The syntax graph of the biological pattern language. Complex patterns can be generated by combining simple patterns.

Our approach is compositional, meaning that by combining each pattern entity using Boolean operators (i.e. AND, OR, IMPLY etc.), we can construct more complex patterns (figure 5), which can then be analysed using a suitable model checking tool.

### 5.1. Selecting the fastest tool for verification of property patterns

There are a number of tools that can be used in verification. In [39], we have showed that the ‘performance of each tool significantly varies according to’ (i) type of biological property, as each pattern can ‘involve different computational processes on the network working at different levels of complexity (e.g. searching some nodes, or all nodes etc.)’, and (ii) ‘the topological features and characteristics of the underlying network of a given model (e.g. number of vertices and edges, graph density, graph degree etc.)’.

In order to facilitate choosing the right tool for different property patterns, we have developed a software system [39] that predicts the fastest tool for a given biomodel and desired property pattern. Our prediction models work with 90% accuracy, implying a ‘huge performance gain compared with the random selection of tools, as choosing the most efficient model checker will result in significantly less verification time’ (hours in some cases) [39].

## 6. A verification methodology for biology

Although model checking has been used extensively in systems biology, it has mainly been performed by computer scientists. Model checking is a very complex process for biologists because it requires a comprehensive technical background on verification and reasonable familiarity with both modelling and property specification languages [15].

In this section, we provide guidance and methodology on how to use model checking for the formal analysis of biological systems and biomolecular networks. This methodology is mainly based on our review of the application of formal verification in systems biology. We believe that this guidance will facilitate the verification process for biologists who do not have the background of ‘formal methods’.

(i) **Informal system description**. The first step is to write an informal description of the system. This should include all the necessary system dynamics. A good informal model of the system should be *clear*, *simple*, *traceable*, *truthful* (to the system), and *extensible* and *reusable* [185]. A good system model helps us answer important questions regarding its behaviour.
(ii) **Informal requirements**. The next step is to decide what you want to know about this system, and which information you want to infer about system properties. A good strategy is to write behaviour requirements in the form of informal queries [186]. Structured queries in a natural language will help to formulate them in a formal syntax.
(iii) **Formal modelling**. Depending on the characteristics described in the informal model, a suitable modelling approach should be selected [1]. For example, if the system comprises a set of kinetic rules, we can use either ODEs or ruled-based systems. In most cases, there is no need to code the model in the syntax that a model checker accepts as an input. Many software suites used in systems biology can export models for model checkers. Therefore, we can use a high-level description language supported by a software suite.
(iv) **Formal specification**. Depending on the type of informal queries, we will either need to express them in a temporal or a probabilistic logic. However, property specification is a very difficult and error-prone task for non-experts [107,177]. We therefore suggest to use a formal specification language which has a tool support. Our tool presented in [15] has such a support for non-experts. This tool automatically converts the pattern language illustrated in figure 5 into the formal specification languages required by model checkers.
(v) **Formal verification**. Based on the modelling formalism and specification language, a suitable model checking tool is selected to query the property in question. An integrated workbench will be more suitable for this purpose, as it provides support for various tools and performs the necessary translations automatically.
(vi) **Methods/techniques to improve efficiency**. As stated in §2, model checking suffers from the *state explosion* problem. This is especially the case for large models such as biological systems. There is no remedy to eliminate this issue entirely, but researchers have suggested some methods to improve the efficiency of model checking:
  — *Population-based approach*. This is a reduction method that models the total number of molecules of each species, rather than modelling the state of each individual component [182].
  — *Discrete intervals for concentration levels*. Modelling the exact concentration levels has a significant effect on the state space. A good strategy, where applicable, is therefore to define a set of intervals, e.g. ‘low-level, medium-level and high-level’, representing concentration levels [40].
  — *Statistical model checking*. Standard model checking explores the entire state space, which is not feasible for large biological systems. An alternative approach is to use statistical model checking, where the verification of a property is considered on finitely many simulations instead of *all* behaviours. Statistical model checking provides a significant advantage in terms of performance and efficiency over standard model checking [89].
  — *Predicting the fastest tool*. As discussed in §5, the property type, model features and characteristics can significantly affect the verification performance. A good strategy is to use the software system presented in [39], which predicts the fastest tool based on a given biomodel and biological property, as this could provide significant gain in the verification process.

An application of this methodology to a synthetic biology case study is presented in electronic supplementary material, §2.

## 7. Discussions

Almost all studies reviewed in this paper are within the scope of systems biology. Although systems biology is important for studying existing systems, we also want to study systems that do not exist in nature. To this end, synthetic biology, an emerging field of biology featuring the design and engineering of new biological systems that do not exist in nature, is developing rapidly. As DNA sequencing and synthesis technologies become cheaper and more easily accessible [187], the scale and complexity of engineering biology projects are set to grow. Hence, it becomes more important to ensure that what the system is meant to do is actually what the models say it would do. That is, it becomes more important to formally verify engineered biological systems to ensure that they meet design requirements.

Along with rapid developments in this field, software suites for modelling and analysing synthetic biology have boosted over the last couple of years. Among these software tools are Eugene [188], gec [189], Proto [190] and Tinkercell [191]. However, our survey has shown that there is almost no effort to support the verification of synthetic biology-related tools and methods. For example, none of these tools integrate or provide support for model checking. This is mainly due to the fact that synthetic biology introduces new challenges, difficult for existing model checking approaches to cope with. As an initial step to tackle these challenges, we have proposed a novel methodology and initial set of tools [15,39,73,98,164]. We are currently developing a new software platform that fully implements this methodology, which has been defined based on the extensive review presented in this paper.

## 8. Conclusion

In this paper, we reviewed the most frequently used modelling approaches for computational biology and also compared model checking tools that were applied to analyse biological systems and biochemical networks. There is growing interest in using model checking in both systems and synthetic biology, as this approach provides a deeper mechanistic understanding of the underlying biology. Numerous biological systems have been analysed, from biological pathways to genetic bio-devices.

Our survey showed that most related studies focus on systems biology. Although there have been a few case studies, their applications in synthetic biology are quite limited. This is mainly due to the fact that synthetic biology introduces new challenges, difficult for existing model checking approaches to cope with.

In future research, we are currently working on an integrative perspective combining different model checking approaches based on different property types, automatically selecting the fastest tool given the model and property pattern, and the use of some natural language patterns to express various properties. This approach will make the use of the model checking methods very efficient, effective, and easy to use, even for non-experts in formal methods, which is coherent with the engineering viewpoint in synthetic biology.

## Data Availability

The data are provided in electronic supplementary material [192].

## References

[1] Fisher J, Henzinger TA. 2007 Executable cell biology. Nat. Biotechnol. **25**, 1239-1249. (10.1038/nbt1356) 17989686

[2] Priami C. 2009 Algorithmic systems biology. Commun. ACM **52**, 80-88. (10.1145/1506409.1506427)

[3] Calder M, Gilmore S, Hillston J. 2005 Automatically deriving ODEs from process algebra models of signalling pathways. In *Proc. of CMSB 2005*, pp. 204–215.

[4] Feiglin A, Hacohen A, Sarusi A, Fisher J, Unger R, Ofran Y. 2012 Static network structure can be used to model the phenotypic effects of perturbations in regulatory networks. Bioinformatics **28**, 2811-2818. (10.1093/bioinformatics/bts517) 22923292

[5] Talcott C. 2006 Symbolic modeling of signal transduction in pathway logic. In *Proc. of the 38th Conf. on Winter Simulation, WSC ’06, Monterey, CA, 3–6 December*, pp. 1656–1665. Winter Simulation Conference.

[6] Deutsch A, Dormann S. 2004 Cellular automata modeling of biological pattern formation. Berlin, Germany: Springer.

[7] Aniba MR, Thompson JD. 2010 Knowledge based expert systems in Bioinformatics. In *Expert Systems* (ed. P Vizureanu), ch. 10. Rijeka, Croatia: IntechOpen.

[8] Bashirov R, Mehraei M. 2017 Identifying targets for gene therapy of β-globin disorders using quantitative modeling approach. Inf. Sci. **397–398**, 37-47. (10.1016/j.ins.2017.02.053)

[9] Colomer MA, Margalida A, Pérez-Jiménez MJ. 2013 Population dynamics P system (PDP) models: a standardized protocol for describing and applying novel bio-inspired computing tools. PLoS ONE **8**, 1-13. (10.1371/journal.pone.0060698) PMC3622025 23593284

[10] Garcia-Quismondo M, Levin M, Lobo D. 2017 Modeling regenerative processes with membrane computing. Inf. Sci. **381**, 229-249 (10.1016/j.ins.2016.11.017)

[11] Bauer AL, Beauchemin CAA, Perelson AS. 2009 Agent-based modeling of host–pathogen systems: the successes and challenges. Inf. Sci. **179**, 1379-1389. (10.1016/j.ins.2008.11.012) PMC2731970 20161146

[12] Soheilypour M, Mofrad M. 2018 Agent-based modeling in molecular systems biology. BioEssays **40**, 1800020. (10.1002/bies.201800020) 29882969

[13] Bernot G, Comet J-P, Richard A, Guespin J. 2004 Application of formal methods to biological regulatory networks: extending Thomas’ asynchronous logical approach with temporal logic. J. Theor. Biol. **229**, 339-347. (10.1016/j.jtbi.2004.04.003) 15234201

[14] Laubenbacher RC. 2007 Modeling and simulation of biological networks. In *Proc. of Symposia in Applied Mathematics*, vol. 64, p. 151. American Mathematical Society.

[15] Konur S, Gheorghe M. 2015 A property-driven methodology for formal analysis of synthetic biology systems. *IEEE/ACM Trans. Comput. Biol. Bioinform.* **12**, 360–371.

[16] Clarke EM, Grumberg O, Peled DA. 1999 Model checking. Cambridge, MA: MIT Press.

[17] Konur S. 2006 A decidable temporal logic for events and states. In *13th Int. Symp. on Temporal Representation and Reasoning (TIME’06), Budapest, Hungary, 15–17 June*, pp. 36–41. IEEE.

[18] Konur S. 2008 An interval logic for natural language semantics. In *Proc. of the 7th Conf. on Advances in Modal Logic, Nancy, France, 9–12 September 2008*, pp. 177–191. CSLI Publications.

[19] Konur S. 2010 Real-time and probabilistic temporal logics: an overview. *CoRR*, abs/1005.3200.

[20] Konur S. 2010 A survey on temporal logics. *CoRR*, abs/1005.3199.

[21] Konur S. 2011 An event-based fragment of first-order logic over intervals. J. Logic, Lang. Inf. **20**, 49-68. (10.1007/s10849-010-9126-5)

[22] Konur S. 2013 A survey on temporal logics for specifying and verifying real-time systems. Front. Comput. Sci. **7**, 370-403. (10.1007/s11704-013-2195-2)

[23] Konur S. 2014 Specifying safety-critical systems with a decidable duration logic. Sci. Comput. Program. **80**, 264-287. (10.1016/j.scico.2013.07.012)

[24] Alur R, McMillan K, Peled D. 2000 Model-checking of correctness conditions for concurrent objects. Inf. Comput. **160**, 167-188. (10.1006/inco.1999.2847)

[25] Yabandeh M. 2011 Model checking of distributed algorithm implementations. PhD thesis, École Polytechnique Fédérale de Lausanne, Switzerland.

[26] Konur S, Fisher M. 2011 Formal analysis of a VANET congestion control protocol through probabilistic verification. In *Proc. of the 73rd IEEE Vehicular Technology Conf., VTC Spring 2011, 15*–*18 May 2011, Budapest, Hungary*, pp. 1–5. IEEE.

[27] Konur S. 2014 Towards light-weight probabilistic model checking. J. Appl. Math. **2014**, 15. (10.1155/2014/814159)

[28] Abbink H et al. 2004 Automated support for adaptive incident management. In *Proc. of ISCRAM’04, Brussels, Belgium*, pp. 153–170.

[29] Konur S, Fisher M, Schewe S. 2013 Combined model checking for temporal, probabilistic, and real-time logics. Theor. Comput. Sci. **503**, 61-88. (10.1016/j.tcs.2013.07.012)

[30] Arapinis M et al. 2009 Towards the verification of pervasive systems. *Electron. Commun. Eur. Assoc. Softw. Sci. Technol.* **22**. (10.14279/tuj.eceasst.22.315)

[31] Konur S, Fisher M, Dobson S, Knox S. 2014 Formal verification of a pervasive messaging system. Formal Aspects Comput. **26**, 677-694. (10.1007/s00165-013-0277-4)

[32] Konur S, Fisher M. 2015 A roadmap to pervasive systems verification. Knowl. Eng. Rev. **30**, 324-341. (10.1017/S0269888914000228)

[33] Konur S, Dixon C, Fisher M. 2010 Formal verification of probabilistic swarm behaviours. In *Swarm Intelligence* (eds M Dorigo *et al.*), vol. 6234 of *Lecture notes in computer science*, pp. 440–447. Berlin, Germany: Springer.

[34] Konur S, Dixon C, Fisher M. 2012 Analysing robot swarm behaviour via probabilistic model checking. Robot. Auton. Syst. **60**, 199-213. (10.1016/j.robot.2011.10.005)

[35] Camci F, Eker OF, Baskan S, Konur S. 2016 Comparison of sensors and methodologies for effective prognostics on railway turnout systems. Proc. Inst. Mech. Eng., Part F: J. Rail Rapid Transit **230**, 24-42. (10.1177/0954409714525145)

[36] Lefticaru R, Konur S, Yildirim Ü, Uddin A, Campean F, Gheorghe M. 2017 Towards an integrated approach to verification and model-based testing in system engineering. In *The Int. Workshop on Engineering Data- & Model-driven Applications (EDMA-2017), Exeter, UK, 21–23 June*, pp. 131–138. IEEE.

[37] Lefticaru R, Bakir ME, Konur S, Stannett M, Ipate F. 2018 Modelling and validating an engineering application in kernel P systems. In *Membrane computing* (eds M Gheorghe, G Rozenberg, A Salomaa, C Zandron), pp. 183–195. Cham, Switzerland: Springer International Publishing.

[38] Bakir ME, Gheorghe M, Konur S, Stannett M. 2017 Comparative analysis of statistical model checking tools. In *Membrane Computing* (eds A Leporati, G Rozenberg, A Salomaa, C Zandron), pp. 119–135, Cham, Switzerland: Springer International Publishing.

[39] Bakir ME, Konur S, Gheorghe M, Krasnogor N, Stannett M. 2018 Automatic selection of verification tools for efficient analysis of biochemical models. Bioinformatics **34**, 3187-3195. (10.1093/bioinformatics/bty282) 29688313 PMC6137970

[40] Heiner M, Gilbert D, Donaldson R. 2008 Petri nets for systems and synthetic biology. In *Formal methods for computational systems biology*, vol. 5016 of *Lecture notes in computer science*, pp. 215–264. Berlin, Germany: Springer.

[41] Heath J, Kwiatkowska M, Norman G, Parker D, Tymchyshyn O. 2008 Probabilistic model checking of complex biological pathways. Theor. Comput. Sci. **319**, 239-257. (10.1016/j.tcs.2007.11.013)

[42] Saeed MT, Ahmad J, Baumbach J, Pauling J, Shafi A, Paracha RZ, Hayat A, Ali A. 2018 Parameter estimation of qualitative biological regulatory networks on high performance computing hardware. BMC Syst. Biol. **12**, 146. (10.1186/s12918-018-0670-y) 30594246 PMC6311083

[43] Eker S, Knapp M, Laderoute K, Lincoln P, Meseguer J, Sonmez K. 2002 Pathway logic: symbolic analysis of biological signaling. In *Proc. of the Pacific Symp. on Biocomputing*, pp. 400–412.

[44] Streck A, Thobe K, Siebert H. 2015 Analysing cell line specific EGFR signalling via optimized automata based model checking. In *Computational methods in systems biology* (eds O Roux, J Bourdon), pp. 264–276, Cham, Switzerland: Springer International Publishing.

[45] Clarke EM, Faeder JR, Langmead CJ, Harris LA, Jha SK, Legay A. 2008 Statistical model checking in Biolab: applications to the automated analysis of T-cell receptor signaling pathway. In *Proc. of the 6th Int. Conf. on Computational Methods in Systems Biology, CMSB’08, Rostock, Germany, 12–15 October*, pp. 231–250. New York, NY: Springer.

[46] Abou-Jaoudé W, Monteiro PT, Naldi A, Grandclaudon M, Soumelis V, Chaouiya C, Thieffry D. 2015 Model checking to assess t-helper cell plasticity. Front. Bioeng. Biotechnol. **2**, 86. (10.3389/fbioe.2014.00086) 25674559 PMC4309205

[47] Sedghamiz H, Morris M, Craddock TJA, Whitley D, Broderick G. 2019 Bio-modelchecker: using bounded constraint satisfaction to seamlessly integrate observed behavior with prior knowledge of biological networks. Front. Bioeng. Biotechnol. **7**, 48. (10.3389/fbioe.2019.00048) 30972331 PMC6443719

[48] Khalid A. 2020 Biometa: a multiple specification parameter estimation system for stochastic biochemical models. *arXiv*. (10.48550/arXiv.2001.03781)

[49] Romero-Campero FJ, Gheorghe M, Bianco L, Pescini D, Pérez-Jiménez MJ, Ceterchi R. 2006 Towards probabilistic model checking on P systems using Prism. In *Membrane computing*, vol. 4361 of *Lecture notes in computer science*, pp. 477–495. Berlin, Germany: Springer.

[50] Shukla A, Bhattacharyya A, Kuppusamy L, Srivas M, Thattai M. 2017 Discovering vesicle traffic network constraints by model checking. PLoS ONE **12**, 1-15. (10.1371/journal.pone.0180692) PMC5500374 28683137

[51] Calzone L, Chabrier-Rivier N, Fages F, Soliman S. 2006 Machine learning biochemical networks from temporal logic properties. In *Transactions on computational systems biology VI*, vol. 4220 of *Lecture notes in computer science*, pp. 68–94. Berlin, Germany: Springer.

[52] Traynard P, Fages F, Soliman S. 2015 Model-based investigation of the effect of the cell cycle on the circadian clock through transcription inhibition during mitosis. In *Computational methods in systems biology* (eds O Roux, J Bourdon), pp. 208–221. Cham, Switzerland: Springer International Publishing.

[53] Mitra ED, Suderman R, Colvin J, Ionkov A, Hu A, Sauro HM, Posner RG, Hlavacek WS. 2019 Pybionetfit and the biological property specification language. iScience **19**, 1012-1036. (10.1016/j.isci.2019.08.045) 31522114 PMC6744527

[54] Hall BA, Fisher J. 2020 Constructing and analyzing computational models of cell signaling with biomodelanalyzer. Curr. Prot. Bioinformatics **69**, e95. (10.1002/cpbi.95) 32078258

[55] Mahmoud EA, Herajy M, Ziedan IE, Shehata HI. 2023 Formal verification confirms the role of p53 protein in cell fate decision mechanism. Theory Biosci. **142**, 29-45. (10.1007/s12064-022-00381-x) 36510032 PMC9925526

[56] Traynard P, Fauré A, Fages F, Thieffry D. 2016 Logical model specification aided by model-checking techniques: application to the mammalian cell cycle regulation. Bioinformatics **32**, i772-i780. (10.1093/bioinformatics/btw457) 27587700

[57] Naldi A, Hernandez C, Abou-Jaoudé W, Monteiro PT, Chaouiya C, Thieffry D. 2018 Logical modeling and analysis of cellular regulatory networks with GINsim 3.0. Front. Physiol. **9**, 646. (10.3389/fphys.2018.00646) 29971008 PMC6018412

[58] Wei O, Guo Z, Niu Y, Liao W. 2017 Model checking optimal finite-horizon control for probabilistic gene regulatory networks. BMC Syst. Biol. **11**, 75-88. (10.1186/s12918-017-0481-6) 29297345 PMC5751526

[59] Liu B, Gyori BM, Thiagarajan PS. 2019 Statistical model checking-based analysis of biological networks. Cham, Switzerland: Springer International Publishing, pp. 63-92.

[60] Klarner H, Siebert H, Nee S, Heinitz F. 2020 Basins of attraction, commitment sets and phenotypes of boolean networks. IEEE/ACM Trans. Comput. Biol. Bioinf. **17**, 1115-1124. 10.1109/TCBB.2018.2879097 30575543

[61] Konur S, Gheorghe M, Dragomir C, Ipate F, Krasnogor N. 2014 Conventional verification for unconventional computing: a genetic XOR gate example. Fundam. Inform. **134**, 97-110. (10.3233/FI-2014-1093)

[62] Konur S, Gheorghe M, Dragomir C, Mierlă L, Ipate F, Krasnogor N. 2015 Qualitative and quantitative analysis of systems and synthetic biology constructs using P systems. ACS Synth. Biol. **4**, 83-92. (10.1021/sb500134w) 25090609

[63] Gilbert D, Heiner M, Ghanbar L, Chodak J. 2019 Spatial quorum sensing modelling using coloured hybrid Petri nets and simulative model checking. BMC Bioinf. **20**, 173. (10.1186/s12859-019-2690-z) PMC6471779 30999841

[64] Cifuentes-Fontanals L, Tonello E, Siebert H. 2022 Control in boolean networks with model checking. Front. Appl. Math. Stat. **8**, 838546. (10.3389/fams.2022.838546)

[65] Boemo MA, Lucas AE, Turberfield AJ, Cardelli L. 2016 The formal language and design principles of autonomous DNA walker circuits. ACS Synth. Biol. **5**, 878-884. (10.1021/acssynbio.5b00275) 27114350

[66] Zhu W, Han YJ, Zhou Q. 2019 Performing CTL model checking via DNA computing. Soft Comput. **23**, 3945-3963. (10.1007/s00500-018-3314-7)

[67] Li B, Mackenzie N, Shirt-Ediss B, Krasnogor N, Zuliani P. 2022 Modelling and optimisation of a DNA stack nano-device using probabilistic model checking. In *28th Int. Conf. on DNA Computing and Molecular Programming (DNA 28)* (eds TE Ouldridge, SFJ Wickham), vol. 238 of *Leibniz Int. Proc. in Informatics (LIPIcs)*, pages 5:1–5:22, Dagstuhl, Germany: Schloss Dagstuhl – Leibniz-Zentrum für Informatik.

[68] Legay A, Sedwards S, Traonouez L-M. 2016 Plasma lab: a modular statistical model checking platform. In *Leveraging applications of formal methods, verification and validation: foundational techniques* (eds T Margaria, B Steffen), pp. 77–93. Cham, Switzerland: Springer International Publishing.

[69] Beneš N, Brim L, Pastva S, Šafránek D. 2019 Model checking approach to the analysis of biological systems, pp. 3-35. Cham, Switzerland: Springer Nature.

[70] Sanassy D, Fellermann H, Krasnogor N, Konur S, Mierlă L, Gheorghe M, Ladroue C, Kalvala S. 2014 Modelling and stochastic simulation of synthetic biological Boolean gates. In *16th IEEE Int. Conf. on High Performance Computing and Communications, Paris, France, 20–22 August*, pp. 404–408. IEEE. 10.1021/acssynbio.1c00143 34339602

[71] Gheorghe M, Konur S, Ipate F. 2017 Kernel P systems and stochastic P systems for modelling and formal verification of genetic logic gates. Cham, Switzerland: Springer International Publishing, pp. 661-675.

[72] Konur S, Fellermann H, Marian Mierla L, Sanassy D, Ladroue C, Kalvala S, Gheorghe M, Krasnogor N. 2017 An integrated in silico simulation and biomatter compilation approach to cellular computation. Cham, Switzerland: Springer International Publishing, pp. 655-676.

[73] Konur S et al. 2021 Toward full-stack *in silico* synthetic biology: integrating model specification, simulation, verification, and biological compilation. ACS Synth. Biol. **10**, 1931-1945. (10.1021/acssynbio.1c00143) 34339602

[74] Bogomolov S, Schilling C, Bartocci E, Batt G, Kong H, Grosu R. 2015 Abstraction-based parameter synthesis for multiaffine systems. In *Hardware and software: verification and testing* (ed. N Piterman), pp. 19–35. Cham, Switzerland: Springer International Publishing.

[75] Neupane T, Zhang Z, Madsen C, Zheng H, Myers CJ. 2019 Approximation techniques for stochastic analysis of biological systems, pp. 327-348. Cham, Switzerland: Springer International Publishing.

[76] Materi W, Wishart DS. 2007 Computational systems biology in cancer: modeling methods and applications. Gene Regul. Syst. Biol. **1**, 91-110. (10.1177/117762500700100010) PMC2759135 19936081

[77] Tenazinha N, Vinga S. 2011 A survey on methods for modeling and analyzing integrated biological networks. IEEE/ACM Trans. Comput. Biol. Bioinf. **8**, 943-958. (10.1109/TCBB.2010.117) 21116043

[78] Priami C. 2012 Algorithmic systems biology – computer science propels systems biology. In *Handbook of natural computing*, pp. 1835–1862. Berlin, Germany: Springer.

[79] Bartocci E, Lió P. 2016 Computational modeling, formal analysis, and tools for systems biology. PLoS Comput. Biol. **12**, 1-22. (10.1371/journal.pcbi.1004591) PMC4721667 26795950

[80] Wang Q. 2016 *Formal methods for biological systems: languages, algorithms, and applications*. Technical Report CMU-CS-16-129, School of Computer Science, Carnegie Mellon University.

[81] Hajnal M. 2018 Formal methods for model selection in systems biology. Master's thesis, Masaryk University, Brno, Czech Republic.

[82] Carrillo M, Gongora PA, Rosenblueth DA. 2012 An overview of existing modeling tools making use of model checking in the analysis of biochemical networks. Front. Plant Sci. **3**, 155. (10.3389/fpls.2012.00155) 22833747 PMC3400939

[83] Brim L, Ceska M, Safranek D. 2013 Model checking of biological systems. In *Formal methods for dynamical systems*, vol. 7938 of *LNCS*, pp. 63–112. New York, NY: Springer. 10.1371/journal.pone.0094553 PMC3994026 24751941

[84] Fisher J, Piterman N. 2014 Model checking in biology. In *A systems theoretic approach to systems and synthetic biology I: models and system characterizations*, pp. 255–279. New York, NY: Springer.

[85] Zuliani P. 2015 Statistical model checking for biological applications. Int. J. Softw. Tools Technol. Transf. **17**, 527-536. (10.1007/s10009-014-0343-0)

[86] Liò P, Zuliani P (eds). 2019 Automated reasoning for systems biology and medicine, vol. 30, *Computational biology*. New York, NY: Springer.

[87] Cimatti A, Clarke E, Giunchiglia E, Giunchiglia F, Pistore M, Roveri M, Sebastiani R, Tacchella A. 2002 NuSMV version 2: an open source tool for symbolic model checking. In *Proc. of CAV 2002*, vol. 2404 of *LNCS*, pp. 359–364. New York, NY: Springer.

[88] Hinton A, Kwiatkowska M, Norman G, Parker D. 2006 Prism: a tool for automatic verification of probabilistic systems. In *Proc. TACAS*, vol. 3920 of *LNCS*, pp. 441–444. New York, NY: Springer.

[89] Sen K, Viswanathan M, Agha G. 2004 Statistical model checking of black-box probabilistic systems. In *Computer a**ided verification*, vol. 3114 of *Lecture notes in computer science*, pp. 202–215. Berlin, Germany: Springer.

[90] Legay A, Delahaye B, Bensalem S. 2010 Statistical model checking: an overview. In *Runtime verification*, vol. 6418 of *Lecture notes in computer science*, pp. 122–135. New York, NY: Springer.

[91] Pnueli A. 1977 The temporal logic of programs. In *Proc. of the 18th Annual IEEE Symp. on Foundations of Computer Science, 30 September–31 October*, pp. 46–57. IEEE Computer Society Press.

[92] Clarke EM, Emerson EA. 1982 Design and synthesis of synchronization skeletons using branching-time temporal logic. In *Logic of Programs, Workshop*, pp. 52–71. New York, NY: Springer.

[93] Hansson H, Jonsson B. 1994 A logic for reasoning about time and reliability. Formal Aspects Comput. **6**, 102-111. (10.1007/BF01211866)

[94] Ognjanović Z. 2006 Discrete linear-time probabilistic logics: completeness, decidability and complexity. J. Logic Comput. **16**, 257-285. (10.1093/logcom/exi077)

[95] Jha SK, Clarke EM, Langmead CJ, Legay A, Platzer A, Zuliani P. 2009 A Bayesian approach to model checking biological systems. In *Computational methods in systems biology*, volume 5688 of *Lecture notes in computer science*, pp. 218–234. Berlin, Germany: Springer.

[96] Baier C, Haverkort B, Hermanns H, Katoen J. 2003 Model-checking algorithms for continuous-time Markov chains. IEEE Trans. Software Eng. **29**, 524-541. (10.1109/TSE.2003.1205180)

[97] Gunawardena J. 2010 Models in systems biology: the parameter problem and the meanings of robustness. In *Elements of computational systems biology* (eds HM Lodhi, SH Muggleton), pp. 19–47. Hoboken, NJ: John Wiley & Sons, Inc.

[98] Blakes J, Twycross J, Konur S, Romero-Campero FJ, Krasnogor N, Gheorghe M. 2014 Infobiotics workbench: a P systems based tool for systems and synthetic biology. In *Applications of membrane computing in systems and synthetic biology*, vol. 7 of *Emergence, complexity and computation*, pp. 1–41. New York, NY: Springer.

[99] Antoniotti M, Policriti A, Ugel N, Mishra B. 2003 Model building and model checking for biochemical processes. Cell Biochem. Biophys. **38**, 271-286. (10.1385/CBB:38:3:271) 12794268

[100] Batt G et al. 2012 Genetic network analyzer: a tool for the qualitative modeling and simulation of bacterial regulatory networks. In *Bacterial molecular networks*, vol. 804 of *Methods in molecular biology*, pp. 439–462. New York, NY: Springer.

[101] Batt G, Yordanov B, Weiss R, Belta C. 2007 Robustness analysis and tuning of synthetic gene networks. Bioinformatics **23**, 2415-2422. (10.1093/bioinformatics/btm362) 17660209

[102] de Jong H, Geiselmann J, Batt G, Hernandez C, Page M. 2004 Qualitative simulation of the initiation of sporulation in *Bacillus subtilis*. Bull. Math. Biol. **66**, 261-299. (10.1016/j.bulm.2003.08.009) 14871567

[103] Viretta AU, Fussenegger M. 2004 Modeling the quorum sensing regulatory network of human-pathogenic *Pseudomonas aeruginosa*. Biotechnol. Prog. **20**, 670-678. (10.1021/bp034323l) 15176867

[104] Batt G, Ropers D, De Jong H, Geiselmann J, Mateescu R, Page M, Schneider D. 2005 Validation of qualitative models of genetic regulatory networks by model checking: analysis of the nutritional stress response in *escherichia coli*. Bioinformatics **21**, 19-28. (10.1093/bioinformatics/bti1048) 15961457

[105] Sepulchre J-A, Reverchon S, Nasser W. 2007 Modeling the onset of virulence in a pectinolytic bacterium. J. Theor. Biol. **244**, 239-257. (10.1016/j.jtbi.2006.08.010) 17005207

[106] Ropers D, de Jong H, Page M, Schneider D, Geiselmann J. 2006 Qualitative simulation of the carbon starvation response in *Escherichia coli*. Biosystems **84**, 124-152. (10.1016/j.biosystems.2005.10.005) 16325332

[107] Monteiro PT, Dumas E, Besson B, Mateescu R, Page M, Freitas AT, de Jong H. 2009 A service-oriented architecture for integrating the modeling and formal verification of genetic regulatory networks. BMC Bioinf. **10**, 1-12. (10.1186/1471-2105-10-450) PMC2813247 20042075

[108] Browning AP, Warne DJ, Burrage K, Baker RE, Simpson MJ. 2020 Identifiability analysis for stochastic differential equation models in systems biology. *bioRxiv*. (10.1101/2020.08.10.245233) PMC7811582 33323054

[109] Lagergren JH, Nardini JT, Michael Lavigne G, Rutter EM, Flores KB. 2020 Learning partial differential equations for biological transport models from noisy spatio-temporal data. Proc. R. Soc. A **476**, 20190800. (10.1098/rspa.2019.0800) 32201481 PMC7069483

[110] Harrington HA, Ho KL, Thorne T, Stumpf MPH. 2012 Parameter-free model discrimination criterion based on steady-state coplanarity. Proc. Natl Acad. Sci. USA **109**, 15 746-15 751. (10.1073/pnas.1117073109) 22967512 PMC3465434

[111] Kauffman SA. 1969 Metabolic stability and epigenesis in randomly constructed genetic nets. J. Theor. Biol. **22**, 437-467. (10.1016/0022-5193(69)90015-0) 5803332

[112] Trairatphisan HP, Mizera A, Pang J, Tantar AA, Schneider J, Sauter T. 2016 Recent development and biomedical applications of probabilistic boolean networks. Cell Commun. Signal. **11**, 46. (10.1186/1478-811X-11-46) PMC3726340 23815817

[113] Thomas R, Kaufman M. 2001 Multistationarity, the basis of cell differentiation and memory. I. structural conditions of multistationarity and other nontrivial behavior. Chaos: Interdiscip. J. Nonlinear Sci. **11**, 170-179. (10.1063/1.1350439) 12779451

[114] Alur R, Henzinger TA. 1999 Reactive modules. Formal Methods Syst. Des. **15**, 7-48. (10.1023/A:1008739929481)

[115] Harel D. 1987 Statecharts: a visual formalism for complex systems. Sci. Comput. Program. **8**, 231-274. (10.1016/0167-6423(87)90035-9)

[116] Efroni S, Harel D, Cohen IR. 2003 Toward rigorous comprehension of biological complexity: modeling, execution, and visualization of thymic T-cell maturation. Genome Res. **13**, 2485-2497. (10.1101/gr.1215303) 14597657 PMC403768

[117] Fisher J, Harel D, Hubbard EJA, Piterman N, Stern MJ, Swerdlin N. 2005 Combining state-based and scenario-based approaches in modeling biological systems. In *Computational methods in systems biology*, vol. 3082 of *Lecture notes in computer science*, pp. 236–241. Berlin, Germany, Springer.

[118] Klop JW. 1992 Term rewriting systems. In *Handbook of logic in computer science* (vol. 2), pp. 1–116. New York, NY: Oxford University Press, Inc.

[119] Faeder JR, Blinov ML, Hlavacek WS. 2009 Rule-based modeling of biochemical systems with Bionetgen. In *Methods in molecular biology, systems biology*, vol. 500 of *Methods in molecular biology*. New York, NY: Humana Press.

[120] Chabrier-Rivier N, Chiaverini M, Danos V, Fages F, Schächter V. 2004 Modeling and querying biomolecular interaction networks. Theor. Comput. Sci. **325**, 25-44. (10.1016/j.tcs.2004.03.063)

[121] Calzone L, Fages F, Soliman S. 2006 BIOCHAM: an environment for modeling biological systems and formalizing experimental knowledge. Bioinformatics **22**, 1805-1807. (10.1093/bioinformatics/btl172) 16672256

[122] Păun G. 2000 Computing with membranes. J. Comput. Syst. Sci. **61**, 108-143. (10.1006/jcss.1999.1693)

[123] Romero-Campero FJ, Gheorghe M, Ciobanu G, Auld JM, Pérez-Jiménez MJ. 2007 Cellular modelling using P systems and process algebra. Prog. Nat. Sci. **17**, 375-383. (10.1080/10020070708541013)

[124] Bakir ME, Ipate F, Konur S, Mierlă L, Niculescu I. 2014 Extended simulation and verification platform for kernel P systems. In *Membrane computing* (eds M Gheorghe, G Rozenberg, A Salomaa, P Sosík, C Zandron), pp. 158–178, Cham, Switzerland: Springer International Publishing.

[125] Sneddon MW, Faeder JR, Emonet T. 2011 Efficient modeling, simulation and coarse-grain of biological complexity with NFsim. Nat. Methods **8**, 177-183. (10.1038/nmeth.1546) 21186362

[126] Danos V, Feret J, Fontana W, Krivine J. 2007 Scalable modelling of biological pathways. In *Asian Symp. on Programming Systems, LNCS 4807, Singapore, 28 November–1 December*, pp. 139–157. Cham, Switzerland: Springer Nature.

[127] Mallavarapu A, Thomson M, Ullian B, Gunawardena J. 2009 Programming with models: modularity and abstraction provide powerful capabilities for systems biology. J. R. Soc. Interface **6**, 257-270. (10.1098/rsif.2008.0205) 18647734 PMC2659579

[128] Natkin S. 1980 *Les reseaux de Petri stochastiques et leur application a levaluation des systemes informatiques*. PhD thesis, CNAM, Paris, France.

[129] Symons FJW. 1980 Introduction to numerical Petri nets, a general graphical model of concurrent processing systems. Australian Telecommun. Res. **14**, 28-33.

[130] Jensen K, Kristensen LM. 2009 Coloured Petri nets: modelling and validation of concurrent systems. Cham, Switzerland: Springer Nature.

[131] Gao Q, Liu F, Tree D, Gilbert D. 2011 Multi-cell modelling using coloured Petri nets applied to planar cell polarity. In *Proc. of the 2nd Int. Workshop on Biological Processes & Petri Nets (BioPPN2011)*, pp. 135–150.

[132] Milner R. 1999 Communicating and mobile systems: π-calculus. Cambridge, UK: Cambridge University Press.

[133] Priami C. 1995 Stochastic *π*-calculus. Comput. J. **38**, 578-589. (10.1093/comjnl/38.7.578)

[134] Calder M, Gilmore S, Hillston J. 2006 Modelling the influence of RKIP on the ERK signalling pathway using the stochastic process algebra PEPA. In *Transactions on computational systems biology VII*, vol. 4230 of *Lecture notes in computer science*, pp. 1–23. Berlin, Germany: Springer.

[135] Calder M, Gilmore S, Hillston J, Vyshemirsky V. 2006 Formal methods for biochemical signalling pathways. In *Formal methods: state of the art and new directions*, pp. 185–215. Berlin, Germany: Springer.

[136] Calder M, Duguid A, Gilmore S, Hillston J. 2006 Stronger computational modelling of signalling pathways using both continuous and discrete-state methods. In *Proc. of CMSB 2006, Trento, Italy, 18–19 October*, vol. 4210 of *LNCS*, pp. 63–77. Springer.

[137] Gerosa L. 2007 Stochastic process algebras as design and analysis framework for synthetic biology modelling. Master’s thesis, University of Trento, Italy.

[138] Ciocchetta F, Hillston J. 2009 Bio-pepa: a framework for the modelling and analysis of biological systems. Theor. Comput. Sci. **410**, 3065-3084. (10.1016/j.tcs.2009.02.037)

[139] Regev A, Panina EM, Silverman W, Cardelli L, Shapiro E. 2004 BioAmbients: an abstraction for biological compartments. Theor. Comput. Sci. **325**, 141-167. (10.1016/j.tcs.2004.03.061)

[140] Dematté L, Priami C, Romanel A. 2008 The Blenx language: a tutorial. In *Formal Methods for Computational Systems Biology, SFM 2008, Bertinoro, Italy, 2–7 June*, number 5054 in LNCS, pp. 123–138. Springer. 10.1093/bib/bbn023 18463130

[141] Bradley JT, Thorne T. 2006 Stochastic process algebra models of a circadian clock. In *Simulation and verification of dynamic systems* (eds DM Nicol, C Priami, HR Nielson, AM. Uhrmacher), vol. 6161 of *Dagstuhl Seminar Proceedings (DagSemProc)*, pp. 1–6, Dagstuhl, Germany: Schloss Dagstuhl – Leibniz-Zentrum für Informatik.

[142] Blossey R, Cardelli L, Phillips A. 2008 Compositionality, stochasticity, and cooperativity in dynamic models of gene regulation. HFSP J **2**, 17-28. (10.2976/1.2804749) 19404450 PMC2640994

[143] Hucka M et al. 2004 Evolving a lingua franca and associated software infrastructure for computational systems biology: the systems biology markup language (SBML) project. Syst. Biol. **1**, 41-53. (10.1049/sb:20045008) 17052114

[144] Cho K-H, Johansson KH, Wolkenhauer O. 2005 A hybrid systems framework for cellular processes. Biosystems **80**, 273-282. (10.1016/j.biosystems.2004.12.002) 15888342

[145] Coveney PV, Fowler PW. 2005 Modelling biological complexity: a physical scientist’s perspective. J. R. Soc. Interface **2**, 267-280. (10.1098/rsif.2005.0045) 16849185 PMC1578273

[146] Tiwari A. 2008 Abstractions for hybrid systems. Formal Methods Syst. Des. **32**, 57-83. (10.1007/s10703-007-0044-3)

[147] Henzinger TA. 1996 The theory of hybrid automata. In *Proc. of the 11th Annual IEEE Symp. on Logic in Computer Science, New Brunswick, NJ, 27–30 July*, pp. 278–292. IEEE.

[148] Matsuno H, Nagasaki M, Miyano S. 2011 Hybrid Petri net based modeling for biological pathway simulation. Nat. Comput. **10**, 1099-1120. (10.1007/s11047-009-9164-6)

[149] Wolfram S. 2002 A new kind of science. Champaign, IL: Wolfram Media.

[150] Ermentrout GB, Edelstein-Keshet L. 1993 Cellular automata approaches to biology. J. Theor. Biol. **160**, 97-133. (10.1006/jtbi.1993.1007) 8474249

[151] Bernardini F, Gheorghe M, Romero-Campero F, Walkinshaw N. 2007 A hybrid approach to modelling biological systems. In *Membrane computing*, vol. 4860 of *Lecture notes in computer science*, pp. 138–159. Berlin, Germany: Springer.

[152] Holzmann GJ. 1997 The model checker Spin. IEEE Trans. Software Eng. **23**, 275-295. (10.1109/32.588521)

[153] Arellano G, Argil J, Azpeitia E, Benitez M, Carrillo M, Gongora P, Rosenblueth DA, Alvarez-Buylla ER. 2011 Antelope: a hybrid-logic model checker for branching-time Boolean GRN analysis. BMC Bioinf. **12**, 1-15. (10.1186/1471-2105-12-490) PMC3316443 22192526

[154] Alur R, Henzinger TA, Mang FYC, Qadeer S, Rajamani SK, Tasiran S. 1998 MOCHA: Modularity in model checking. In *Computer aided verification*, vol. 1427 of *Lecture notes in computer science*, pp. 521–525. Berlin, Germany: Springer.

[155] Bae K, Escobar S, Meseguer J. 2013 Abstract logical model checking of infinite-state systems using narrowing. In *RTA 2013* (ed. F van Raamsdonk), vol. 21 of *Leibniz Int. Proc. in Informatics (LIPIcs)*, pp. 81–96. Schloss Dagstuhl–Leibniz-Zentrum fuer Informatik.

[156] Donaldson R, Gilbert D. 2008 *A Monte Carlo model checker for probabilistic LTL with numerical constraints*. Research Report TR-2008-282, Dept. of Computing Science, University of Glasgow.

[157] Barnat J, Brim L, Safránek D. 2010 High-performance analysis of biological systems dynamics with the DiVinE model checker. Brief. Bioinform. **11**, 301-312. (10.1093/bib/bbp074) 20478855

[158] BioDivine. 2020 BioDivine Tools. See https://sybila.fi.muni.cz/tools.html.

[159] Paterson YZ, Shorthouse D, Pleijzier MW, Piterman N, Bendtsen C, Hall BA, Fisher J. 2018 A toolbox for discrete modelling of cell signalling dynamics. Integr. Biol. **10**, 370-382. (10.1039/C8IB00026C) 29855020

[160] Sadot A, Fisher J, Barak D, Admanit Y, Stern MJ, Hubbard EJA, Harel D. 2008 Toward verified biological models. IEEE/ACM Trans. Comput. Biol. Bioinf. **5**, 223-234. (10.1109/TCBB.2007.1076) 18451431

[161] Harel D, Kugler H, Marelly R, Pnueli A. 2002 Smart play-out of behavioral requirements. In *Formal methods in computer-aided design*, vol. 2517 of *Lecture notes in computer science*, pp. 378–398. Berlin, Germany: Springer.

[162] Burch JR, Clarke EM, McMillan KL, Dill DL, Hwang LJ. 1992 Symbolic model checking: 10E20 states and beyond. Inform. Comput. **98**, 142-170. (10.1016/0890-5401(92)90017-A)

[163] Khalis Z, Comet JP, Richard A, Bernot G. 2009 The SMBioNet method for discovering models of gene regulatory networks. Genes, Genomes Genomics **3**, 15-22.

[164] Konur S, Mierla L, Ipate F, Gheorghe M. 2020 kPWorkbench: a software suit for membrane systems. SoftwareX **11**, 100407. (10.1016/j.softx.2020.100407)

[165] Gheorghe M, Ceterchi R, Ipate F, Konur S. 2017 Kernel P systems modelling, testing and verification – sorting case study. In *Membrane computing* (eds A Leporati, G Rozenberg, A Salomaa, C Zandron), pp. 233–250, Cham, Switzerland: Springer International Publishing.

[166] Ţurlea A, Gheorghe M, Ipate F, Konur S. 2019 Search-based testing in membrane computing. J. Membr. Comput. **1**, 241-250. (10.1007/s41965-019-00027-w)

[167] Bakir ME, Konur S, Gheorghe M, Niculescu I, Ipate F. 2014 High performance simulations of kernel P systems. In *16th IEEE Int. Conf. on High Performance Computing and Communications, 20*–*22 August*, pp. 409–412. IEEE.

[168] Dragomir C, Ipate F, Konur S, Lefticaru R, Mierlă L. 2013 Model checking kernel P systems. In *14th Int. Conf. on Membrane Computing*, vol. 8340 of *LNCS*, pp. 151–172. Berlin, Germany: Springer.

[169] Gheorghe M, Konur S, Ipate F, Mierla L, Bakir ME, Stannett M. 2015 An integrated model checking toolset for kernel P systems. In *Membrane computing*, pp. 153–170. Berlin, Germany: Springer.

[170] Fages F, Soliman S. 2018 On robustness computation and optimization in Biocham-4. In *Computational methods in systems biology* (eds M Češka, D Šafránek), pp. 292–299, Cham, Switzerland: Springer International Publishing.

[171] Schroter C, Schwoon S, Esparza J. 2003 The model-checking kit. In *Applications and theory of Petri nets*, vol. 2679 of *Lecture notes in computer science*, pp. 463–472. Berlin, Germany: Springer.

[172] Garavel H, Lang F, Mateescu R, Serwe W. 2011 CADP 2010: a toolbox for the construction and analysis of distributed processes. In *Tools and algorithms for the construction and analysis of systems*, vol. 6605 of *Lecture notes in computer science*, pp. 372–387. Berlin, Germany: Springer.

[173] Rybinski M, Lula M, Banasik P, Lasota S, Gambin A. 2012 Tav4SB: integrating tools for analysis of kinetic models of biological systems. BMC Syst. Biol. **6**, 25. (10.1186/1752-0509-6-25) 22480273 PMC3495710

[174] David A, Larsen KG, Legay A, Mikucionis M, Poulsen DB, Sedwards S. 2015 Statistical model checking for biological systems. Int. J. Softw. Tools Technol. Transf. **17**, 351-367. (10.1007/s10009-014-0323-4)

[175] Pârvu O, Gilbert D. 2016 A novel method to verify multilevel computational models of biological systems using multiscale spatio-temporal meta model checking. PLoS ONE **11**, 1-43. (10.1371/journal.pone.0154847) PMC4871515 27187178

[176] Alexander C, Ishikawa S, Silverstein M, Jacobson M, Fiksdahl-King I, Angel S. 1977 A pattern language: towns, buildings, construction. Oxford, UK: Oxford University Press.

[177] Dwyer MB, Avrunin GS, Corbett JC. 1999 Patterns in property specifications for finite-state verification. In *Proc. of the 21st Int. Conf. on Software Engineering*, ICSE ’99, pp. 411–420. ACM.

[178] Grunske L. 2008 Specification patterns for probabilistic quality properties. In *Proc. of the 30th Int. Conf. on Software Engineering*, ICSE ’08, pp. 31–40. ACM.

[179] Bellini P, Nesi P, Rogai D. 2009 Expressing and organizing real-time specification patterns via temporal logics. J. Syst. Softw. **82**, 183-196. (10.1016/j.jss.2008.06.041)

[180] Barbuti R, Cataudella S, Maggiolo-Schettini A, Milazzo P, Troina A. 2005 A probabilistic model for molecular systems. Fundam. Inform. **67**, 13-27.

[181] Donaldson R, Calder M. 2012 Modular modelling of signalling pathways and their cross-talk. Theor. Comput. Sci. **456**, 30-50. (10.1016/j.tcs.2012.07.003)

[182] Kwiatkowska M, Norman G, Parker D. 2010 Probabilistic model checking for systems biology. In *Symbolic systems biology* (ed. R Iyengar), pp. 31–59. Burlington, MA: Jones and Bartlett.

[183] Ballarini P, Mardare R, Mura I. 2009 Analysing biochemical oscillation through probabilistic model checking. Electron. Notes Theor. Comput. Sci. **229**, 3-19. (10.1016/j.entcs.2009.02.002)

[184] Lakin M, Parker D, Cardelli L, Kwiatkowska M, Phillips A. 2012 Design and analysis of DNA strand displacement devices using probabilistic model checking. J. R. Soc. Interface **9**, 1470-1485. (10.1098/rsif.2011.0800) 22219398 PMC3367817

[185] Mader AH, Wupper H, Boon M. 2007 *The construction of verification models for embedded systems*. Technical Report TR-CTIT-07-02, Centre for Telematics and Information Technology University of Twente, Enschede, The Netherlands.

[186] Burg JFM. 1997 Linguistic instruments in requirements engineering. Amsterdam, The Netherlands: IOS Press.

[187] Kuhn P, Wagner K, Heil K, Liss M, Netuschil N. 2017 Next generation gene synthesis: from microarrays to genomes. Eng. Life Sci. **17**, 6-13. (10.1002/elsc.201600121) 32624724 PMC6999524

[188] Bilitchenko L, Liu A, Cheung S, Weeding E, Xia B, Leguia M, Anderson JC, Densmore D. 2011 Eugene - a domain specific language for specifying and constraining synthetic biological parts, devices, and systems. PLoS ONE **6**, e18882. (10.1371/journal.pone.0018882) 21559524 PMC3084710

[189] Pedersen M, Phillips A. 2009 Towards programming languages for genetic engineering of living cells. J. R. Soc. Interface **6**(Suppl. 4), S437-S450. (10.1098/rsif.2008.0516.focus) 19369220 PMC2843955

[190] Beal J, Lu T, Weiss R. 2011 Automatic compilation from high-level biologically-oriented programming language to genetic regulatory networks. PLoS ONE **6**, e22490. (10.1371/journal.pone.0022490) 21850228 PMC3151252

[191] Chandran D, Sauro HM. 2012 Hierarchical modeling for synthetic biology. ACS Synthet. Biol. **1**, 353-364. (10.1021/sb300033q) 23651289

[192] Konur S, Gheorghe M, Krasnogor N. 2023 Verifiable biology. Figshare. (10.6084/m9.figshare.c.6619763) PMC10169095 37160165

